# Eosinophil-driven immunopathology exacerbates SARS-CoV-2 pneumonia in an OVA-induced airway allergy mouse model

**DOI:** 10.1128/msphere.00123-26

**Published:** 2026-09-28

**Authors:** Takao Iketani, Naoko Iwata-Yoshikawa, Yusuke Sakai, Nozomi Shiwa-Sudo, Seiya Ozono, Kaya Miyazaki, Tadaki Suzuki, Noriyo Nagata

**Affiliations:** 1 National Institute of Infectious Diseases, Japan Institute for Health Security 739298 , Tokyo, Japan; 2 Cooperative Division of Veterinary Sciences, Graduate School of Agriculture, Tokyo University of Agriculture and Technology 13125, Tokyo, Japan; Capital Medical University Beijing Ditan Hospital, Beijing, China

**Keywords:** SARS-CoV-2, animal model, immunopathology, airway allergy

## Abstract

Asthma is an allergic airway disease characterized by distinct immune environments that can alter host responses to respiratory viral infections; however, its impact on coronavirus disease 2019 (COVID-19) severity remains unclear. To investigate how allergic airway inflammation affects severe acute respiratory syndrome coronavirus 2 (SARS-CoV-2)-induced pneumonia, we used an ovalbumin (OVA)-induced airway allergy mouse model and infected mice with QHmusX, a mouse-passaged SARS-CoV-2 strain that causes severe acute pneumonia. Compared with phosphate-buffered saline-treated controls, OVA-sensitized mice exhibited persistent weight loss, a shift toward prominent alveolar involvement by viral infection, increased inflammatory cell infiltration, and higher histopathological scores, despite comparable viral burden. Enhanced apoptosis and increased numbers of CC10-positive epithelial cells indicated pronounced epithelial injury and repair responses in allergic mice. The post-infection decline in tissue eosinophils, together with histological evidence of eosinophil degranulation and lung cytokine profiling, was associated with amplified inflammatory and granulocyte-associated mediator responses in the airways. To define the functional contribution of eosinophils, we depleted eosinophils using an anti-interleukin-5 monoclonal antibody. Eosinophil depletion improved weight recovery, attenuated lung inflammation, and reduced CC10-associated repair responses without altering viral load. These findings demonstrate that airway allergy exacerbates SARS-CoV-2-induced pneumonia through eosinophil-mediated immunopathology and identify eosinophils as key contributors to disease severity. Targeting eosinophilic inflammation may therefore represent a potential therapeutic strategy for allergic individuals with COVID-19.

**IMPORTANCE:**

Airway allergy profoundly alters host immunity; however, its impact on coronavirus disease 2019 severity remains unresolved. Using an ovalbumin-induced airway allergy mouse model, we show that pre-existing allergic airway inflammation exacerbates severe acute respiratory syndrome coronavirus 2 (SARS-CoV-2)-induced pneumonia despite comparable viral burden. Allergic mice exhibited rapid eosinophil degranulation accompanied by extracellular eosinophil cationic protein deposition, amplified inflammatory mediator responses, and increased epithelial injury. Depletion of eosinophils with anti-interleukin-5 improved weight recovery and lung pathology without measurably affecting viral burden, supporting a pathogenic role for eosinophils in disease exacerbation. These findings indicate that the allergic airway environment can redirect the pulmonary response to SARS-CoV-2 infection and promote immunopathology independently of increased viral replication. The study provides a mechanistic framework for understanding how active allergic inflammation may contribute to severe viral pneumonia and supports further evaluation of eosinophil-targeted therapies in selected clinical contexts.

## INTRODUCTION

Asthma is widely recognized as a risk factor for respiratory viral infections, including respiratory syncytial virus and rhinovirus (1, 2). However, epidemiological studies have produced inconsistent conclusions regarding its association with coronavirus disease 2019 (COVID-19) severity. Several reports indicate that asthma may not increase the risk of severe acute respiratory syndrome coronavirus 2 (SARS-CoV-2) infection or severe disease in adults or children (3, 4). In contrast, nationwide cohort studies in Scotland conducted during the early phase of the pandemic showed that adults and children with an asthma attack or asthma-related hospitalization within the preceding 2 years had a higher risk of COVID-19-related hospitalization, intensive care unit admission, or death than individuals without asthma (5, 6). In the United States, asthma exacerbations were also more frequent among children infected with SARS-CoV-2 during the Omicron wave than during the pre-Delta or Delta waves (7). Conversely, one clinical study reported that patients with asthma and elevated eosinophil counts tended to have lower rates of severe COVID-19 and mortality (8). Collectively, these findings suggest that an allergic immune background influences SARS-CoV-2 pathogenesis in complex, context-dependent ways.

In our previous study using an ovalbumin (OVA)-induced allergic airway inflammation mouse model (9), we demonstrated that allergic airway inflammation skews immune responses toward a Th2-dominant phenotype and markedly redistributes angiotensin-converting enzyme 2 (ACE2)-expressing epithelial cells that are susceptible to SARS-CoV-2 Omicron BA.5 infection. OVA sensitization induced the emergence of ACE2-negative, mucus-secreting goblet cells in the bronchiolar epithelium, accompanied by a reduction in ACE2-expressing cells in the bronchioles and concomitant increase in ACE2-positive cells in the alveolar regions. Consequently, ACE2-expressing target cells became concentrated in the distal airways and alveoli rather than in the proximal bronchiolar epithelium. Consistent with this redistribution, viral infection localized earlier to the alveolar regions and caused greater tissue injury despite lower peak infectious viral titers, with delayed viral RNA clearance and a Th2-skewed inflammatory profile in Omicron BA.5-infected allergic airways.

However, because Omicron BA.5 causes limited lower respiratory tract pathology in BALB/c mice, the extent to which airway allergy affects the development of acute viral pneumonia remained uncertain. To address this gap, we used QHmusX, a mouse-passaged SARS-CoV-2 strain that induces severe acute pneumonia in BALB/c mice (10), thereby enabling detailed evaluation of infection dynamics and tissue injury in an allergic airway environment. Given the central role of eosinophils in asthma pathophysiology and their reported dual functions in antiviral defense and tissue-damaging inflammation, we hypothesized that eosinophils may either exacerbate or ameliorate SARS-CoV-2-induced pneumonia depending on the immune context.

In this study, we examined how airway allergy modifies SARS-CoV-2-induced pneumonia and assessed the functional contribution of eosinophils through anti-interleukin-5 (IL-5)-mediated eosinophil depletion. This approach enabled us to evaluate how eosinophilic inflammation influences disease severity, tissue injury, and epithelial repair responses during acute SARS-CoV-2 infection.

## RESULTS

### Airway allergy enhances disease severity following mouse-passaged SARS-CoV-2 infection

**Fig 1:**
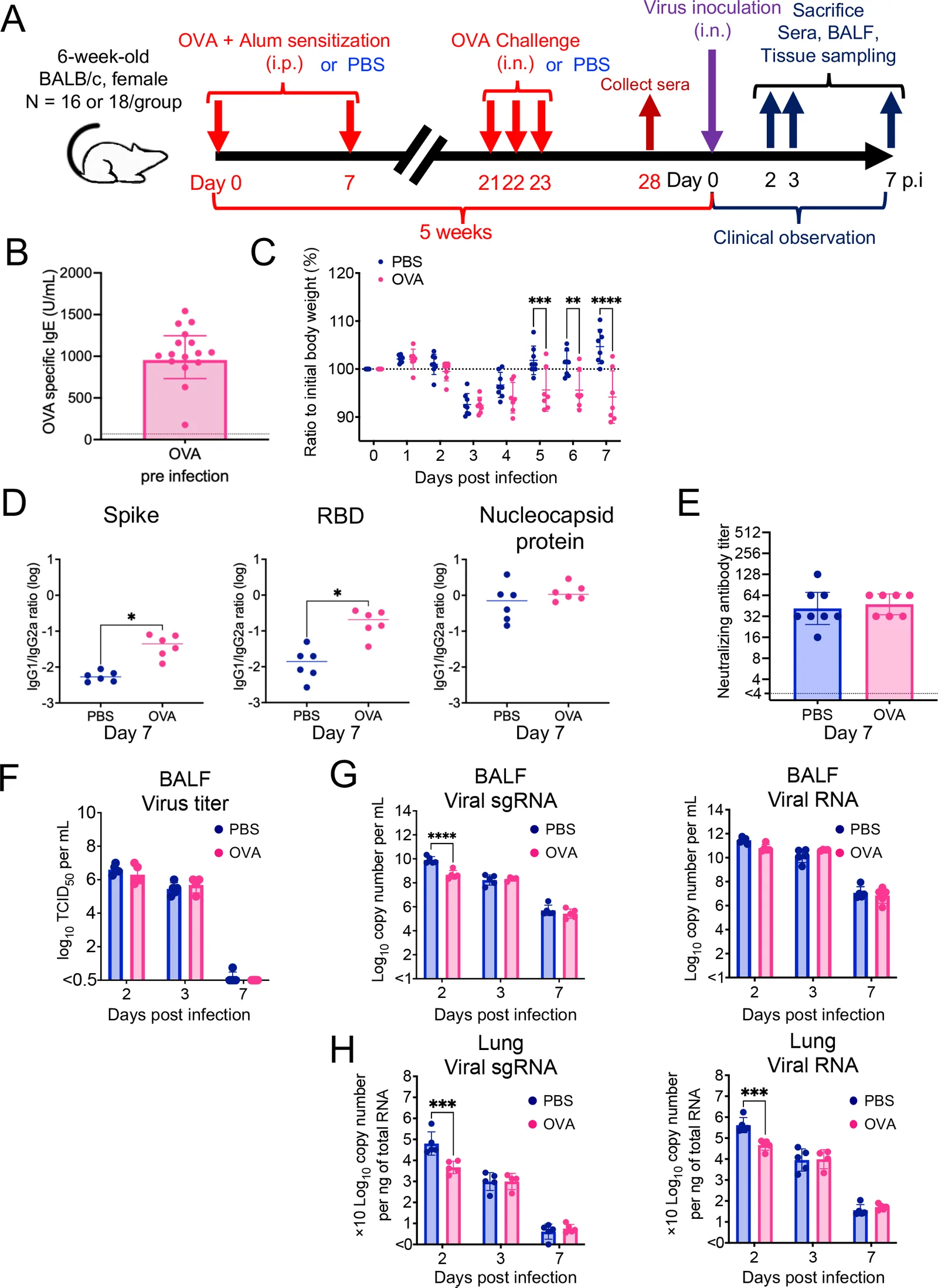
Experimental infection of OVA-induced airway allergy mice with the mouse-adapted SARS-CoV-2 strain QHmusX. (**A**) Schedule of OVA sensitization and challenge of 6-week-old female BALB/c mice. Mice were aged 11 weeks at the time of infection. (**B**) OVA-specific IgE levels in serum were measured 4 weeks after initial sensitization. Each dot represents data from an individual animal in the OVA group (*n* = 16). The dashed line indicates the average level of OVA-specific IgE in PBS-treated mice (*n* = 5; mean ± SD = 67.54 ± 89.16). Error bars indicate the GMT with 95% confidence interval (CI). (**C**) Changes in body weight after experimental infection with QHmusX. Each dot represents data from an animal (*n* = 7 or 8 mice per group). **, *P* < 0.01; ***, *P*  <  0.001; and ****, *P* < 0.0001; two-way ANOVA followed by Šídák’s multiple comparisons test. Error bars indicate the mean  ±  SD. (**D**) Spike-, RBD-, and nucleocapsid-specific IgG1/IgG2a ratios at day 7 p.i. Ratios were calculated from ECL signals obtained using 1:200-diluted serum samples. *P* < 0.05; unpaired two-tailed *t*-test (*n* = 6 mice per group). (**E**) Serum neutralizing antibody titer at 7 days post-infection. No significant difference between the two groups by unpaired *t*-test (*n* = 7 or 8 mice per group). Error bars indicate the GMT with 95% CI. (**F and G**) Bar graphs showing infectious viral titers (**F**) and viral RNA copy number (**G**) in bronchoalveolar lavage fluid (BALF): sgRNA and subgenomic RNA. (**H**) Bar graph showing viral RNA copy number in the right lung lobe. ***, *P* < 0.001 and ****, *P* < 0.0001; two-way ANOVA followed by Šídák’s multiple comparisons test (*n* = 4 or 5 mice per group). Error bars indicate the mean ± SD.

We generated mice with OVA-induced airway allergy (OVA group) and infected them 1 week after the final OVA challenge (Fig. 1A). Before infection, OVA mice exhibited elevated serum OVA-specific IgE levels (Fig. 1B). Following intranasal inoculation with SARS-CoV-2 QHmusX, both phosphate-buffered saline (PBS)-treated controls and OVA mice showed transient weight loss on day 3 post-infection (p.i.). However, five of seven OVA mice failed to regain body weight by day 7, whereas PBS mice recovered progressively (Fig. 1C). By day 7 p.i., SARS-CoV-2-specific IgG1 and/or IgG2a responses against spike, receptor-binding domain (RBD), and nucleocapsid (N) proteins were detected, although response magnitude differed by antigen and group (Fig. S1). Spike-specific IgG1 was detected in both groups, whereas RBD-specific IgG1 remained low in PBS mice and was markedly higher in OVA mice. Accordingly, OVA mice exhibited significantly higher spike- and RBD-specific IgG1 responses than those in PBS mice, whereas IgG2a responses were comparable between groups. Spike- and RBD-specific IgG1/IgG2a ratios were therefore significantly higher in OVA mice, indicating a Th2-biased humoral immune response (Fig. 1D). In contrast, N-specific IgG1 and IgG2a responses increased to similar levels in PBS and OVA mice, resulting in no significant difference in the N-specific IgG1/IgG2a ratio between groups (Fig. S1; Fig. 1D). Despite these antigen-specific differences in IgG subclass responses, neutralizing antibody titers on day 7 were comparable between groups (Fig. 1E).

Infectious viral load in BALF was similar between groups on days 2 and 3 p.i. and was undetectable by day 7 (Fig. 1F). Furthermore, virus-associated RNA levels in BALF and lung tissue followed broadly comparable trajectories. Although overall viral kinetics were similar between groups, PBS mice showed higher BALF subgenomic RNA levels (Fig. 1G) and virus-associated RNA levels in lung tissue (Fig. 1H) on day 2 p.i.

**Fig 2:**
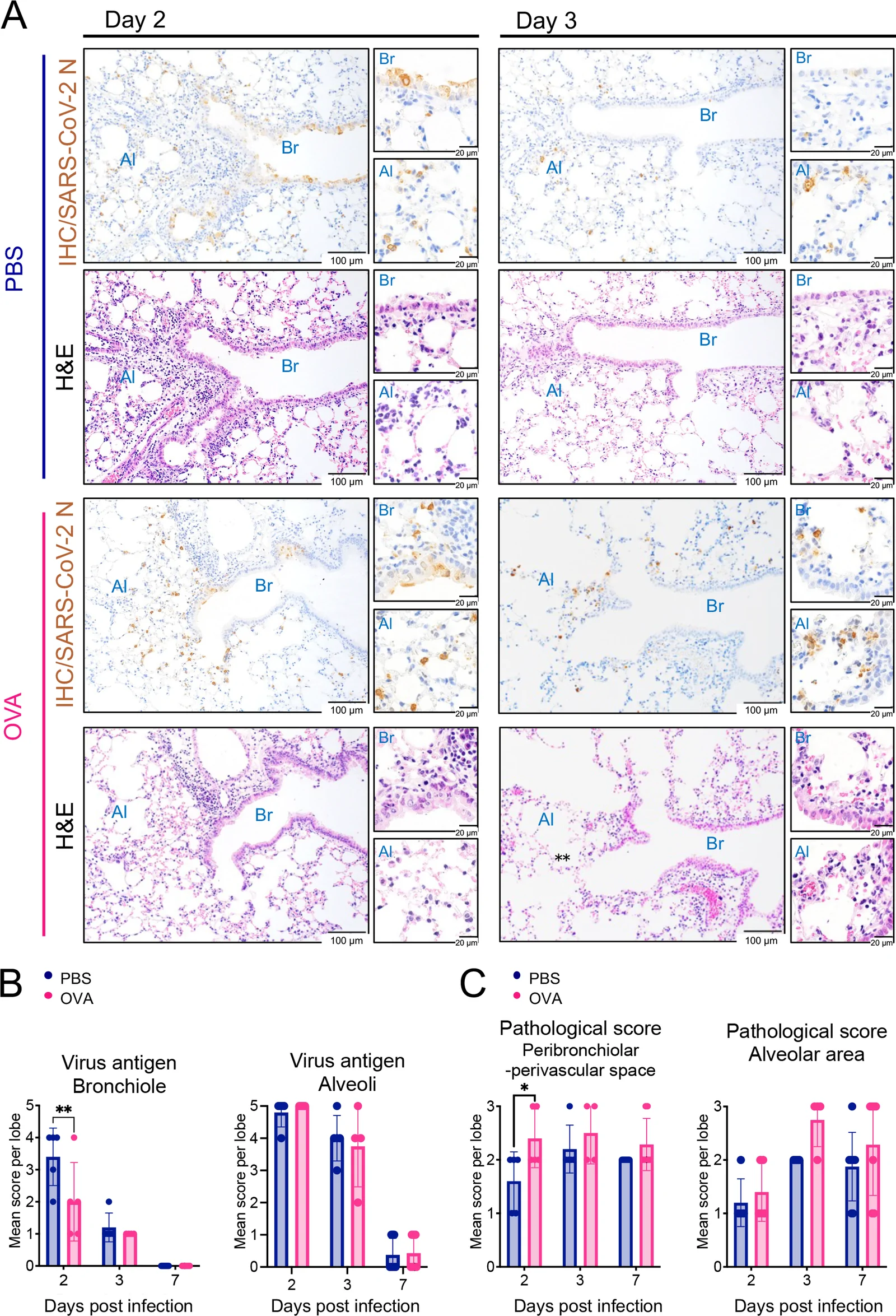
Spatial distribution of SARS-CoV-2 antigens in the lungs of QHmusX-infected mice. (**A**) Representative histopathological findings in the lungs of mice infected with QHmusX (*n* = 4 or 5 mice per group) on days 2 and 3 p.i.. Immunohistochemistry (IHC) was conducted to detect the SARS-CoV-2 N antigen. Al, alveoli; Br, bronchiole. Scale bars, 20 and 100 µm. (**B and C**) Bar graphs show scoring of viral antigen-positive cells (**B**) and pathological lesions (**C**) in different regions of the respiratory tract. *, *P*  <  0.05 and **, *P* < 0.01; two-way ANOVA followed by Šídák’s multiple comparisons test (*n* = 4 or 5 mice per group). Error bars indicate the mean ± SD.

Histological analysis detected viral antigen primarily in bronchioles and alveolar regions in both groups on days 2 and 3 p.i. (Fig. 2A). Quantitative scoring showed higher viral antigen levels in the bronchiolar epithelium of PBS mice on day 2, whereas alveolar antigen levels were comparable between groups (Fig. 2B). In contrast, OVA mice exhibited pronounced inflammatory cell infiltration in the peribronchiolar–perivascular space, resulting in significantly higher pathology scores on day 2 p.i. (Fig. 2C). In the alveolar regions, pathology scores increased more markedly in OVA mice on day 3 p.i. By day 7 p.i., only minimal residual viral antigen was detected in the alveolar regions of two of four mice in each group, whereas inflammatory lesions persisted in both groups (Fig. 2B and C).

**Fig 3:**
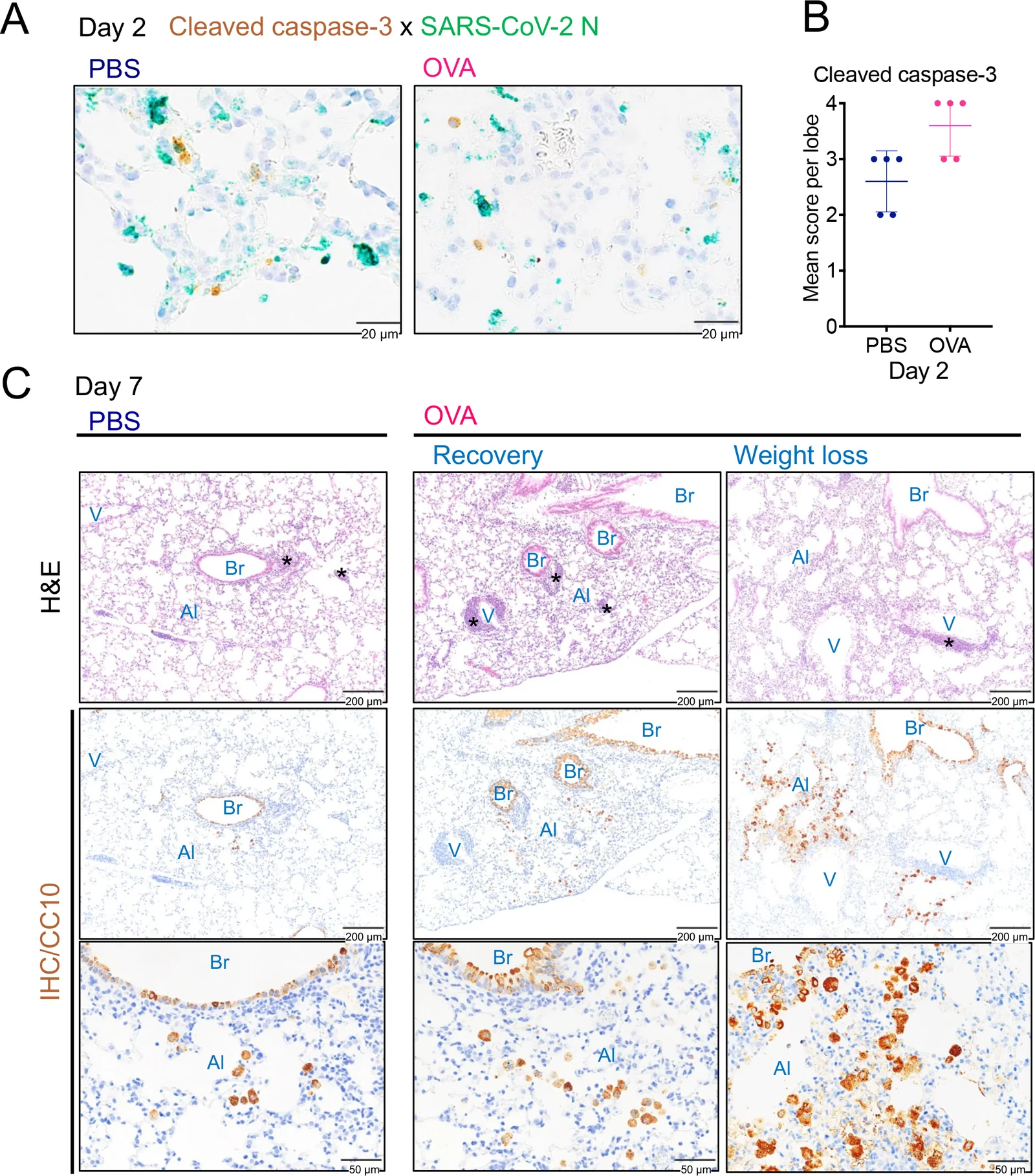
Enhanced pulmonary injury and epithelial damage in OVA-induced airway allergy mice after QHmusX infection. (**A**) Double immunohistochemical staining for caspase-3 (brown) and viral antigen (green) in the alveoli of the PBS and OVA groups at 2 days p.i.. Scale bars, 20 µm. (**B**) Dot plot showing caspase-3 antigen scoring. Five lung lobes were taken from each individual animal, and the caspase-3 antigen score per lobe was assessed under a microscope. Each dot represents the mean value from an individual animal. Error bars indicate the mean ± SD. (**C**) Representative images showing lung pathology in the PBS and OVA groups on day 7 p.i. with QHmusX infection (*n* = 5 mice per group). Recovery: histopathology of a representative animal that recovered its body weight, as shown in Fig. 1C; weight loss, histopathology of a representative animal that continued to lose body weight, as shown in Fig. 1C. Scale bars, 200 µm and 50 µm. H&E, hematoxylin and eosin staining; IHC/CC10, immunohistochemical staining for the CC10 antigen. Asterisks, cell infiltration; Br, bronchiole; V, vessels; Al, alveoli.

To evaluate tissue injury, we quantified cleaved caspase-3-positive apoptotic cells on day 2 p.i. OVA mice showed a trend toward higher apoptotic scores in the alveolar regions than those in PBS mice (Fig. 3A and B; *P* = 0.08, Mann–Whitney *U* test). On day 7 p.i., club cell 10-kDa protein (CC10) immunostaining revealed markedly increased numbers of CC10-positive cells in both bronchiolar and alveolar regions of OVA mice, whereas PBS mice showed only focal, limited increases (Fig. 3C). This increase was particularly evident in mice exhibiting persistent weight loss (Fig. 3C, right panel), suggesting enhanced epithelial repair responses following severe infection. Collectively, these findings indicate that airway allergy predisposes mice to persistent weight loss, exacerbated pulmonary inflammation, and enhanced epithelial injury following QHmusX infection.

### OVA-induced airway allergy mice exhibit eosinophil-associated inflammatory responses following infection

**Fig 4:**
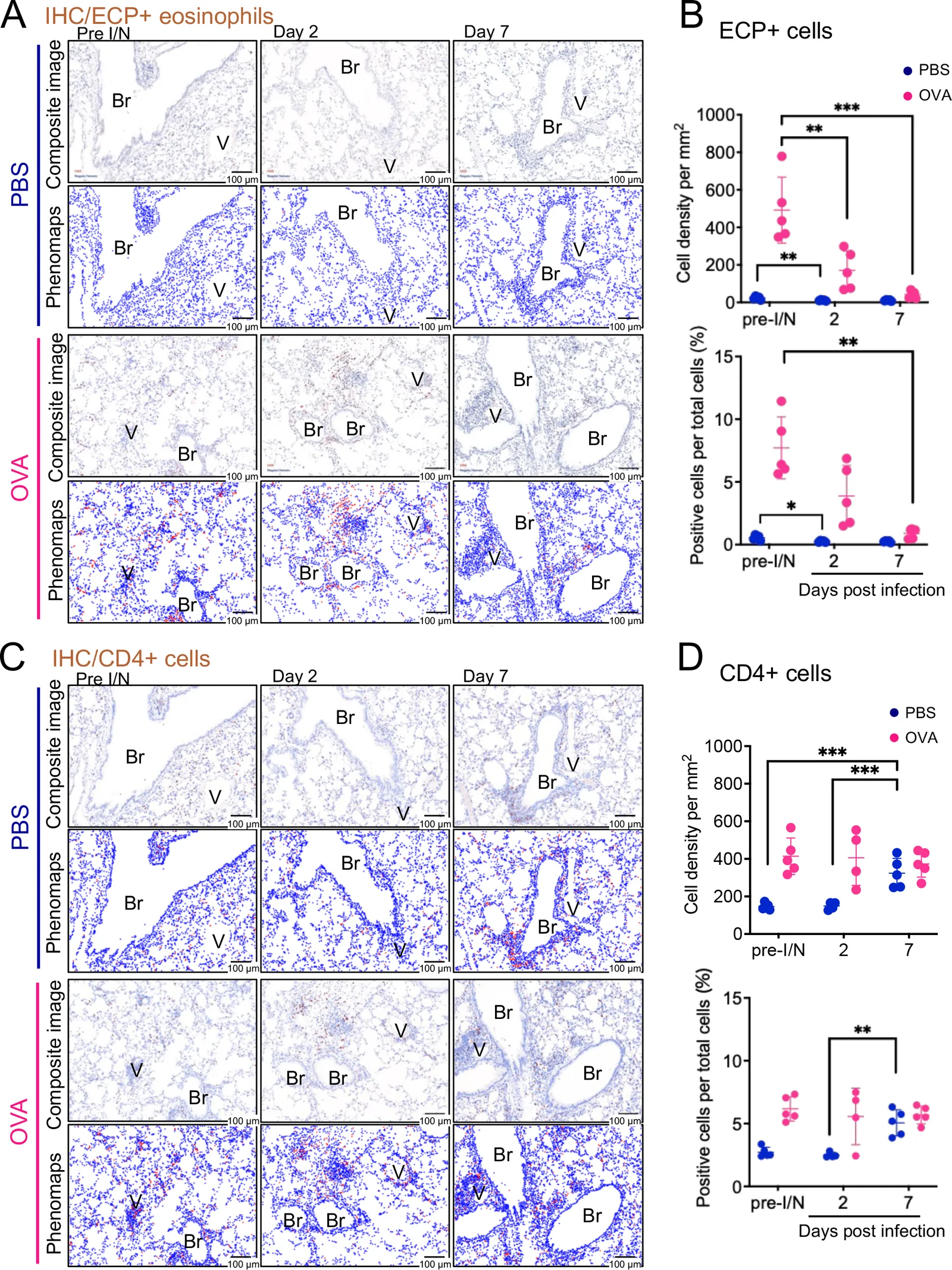
Eosinophil and CD4^+^ T-cell infiltration in the lungs of QHmusX-infected OVA-induced airway allergy mice. (**A and D**) Perivascular and peribronchiolar inflammatory cell infiltration in PBS- and OVA-treated mice. Infiltration with ECP-expressing eosinophils (**A and B**) and CD4^+^ T cells (**C and D**) was evaluated by digital pathology at pre-infection (pre I/N) and p.i. with QHmusX. Cells were counted using the phenotyping software inForm. Representative digital images: composite images are shown in the upper panel, and phenomap images are shown in the lower panel. The images show a region of interest within the lungs. Br, bronchiole; V, vessels. (**B and D**) Graphs showing cell density per mm^2^ and percentage positive cells. The pre-I/N samples were shared with our previous study (9). Each dot represents the mean value per lobe in an individual animal. *, *P* < 0.05; **, *P* < 0.01; and ***, *P* < 0.001; one-way ANOVA followed by Tukey’s multiple comparisons test for cell density or Kruskal–Wallis test followed by Dunn’s multiple comparisons test for percentage positive cells. Error bars indicate the mean  ±  SD.

We next examined the post-infection dynamics of eosinophils and lymphocytes by immunohistochemistry and digital image analysis. Before infection, OVA mice exhibited higher percentages and densities of eosinophil cationic protein (ECP)-positive eosinophils than those in PBS mice (Fig. 4A and B). Eosinophil density declined markedly in OVA mice by days 2 and 7 p.i.; however, both eosinophil density and percentage of eosinophils remained significantly higher than those in PBS mice at all time points, including day 7 p.i. (*P* < 0.01, Mann–Whitney *U* test), indicating that eosinophil levels did not return to baseline levels observed in PBS mice by day 7. CD4^+^ T-cell levels were higher in OVA mice before infection and remained largely unchanged thereafter, whereas PBS mice showed increased CD4^+^ T-cell infiltration by day 7 (Fig. 4C and D).

To further characterize the early eosinophil response to SARS-CoV-2 infection, we examined eosinophil degranulation. In parallel, the temporal dynamics of eosinophils and neutrophils in the peribronchiolar and alveolar regions were quantified using digital image analysis (Fig. S2). ECP+ eosinophils were present in both compartments of OVA mice before infection but decreased after infection, with the most pronounced reduction in the alveolar regions (Fig. S2A). In contrast, myeloperoxidase-positive (MPO+) neutrophils transiently increased in both groups after infection, peaking in the peribronchiolar regions on day 2 in PBS mice and day 3 in OVA mice, whereas alveolar accumulation peaked on day 3 in both groups (Fig. S2B). We additionally performed double immunohistochemical staining for mast cell tryptase and ECP (Fig. S3). Tryptase-positive mast cells were rarely detected, predominantly in peribronchial connective tissue and the pleura, suggesting that mast cells are unlikely to play a major role in the eosinophil-associated immunopathology observed in this model.

**Fig 5:**
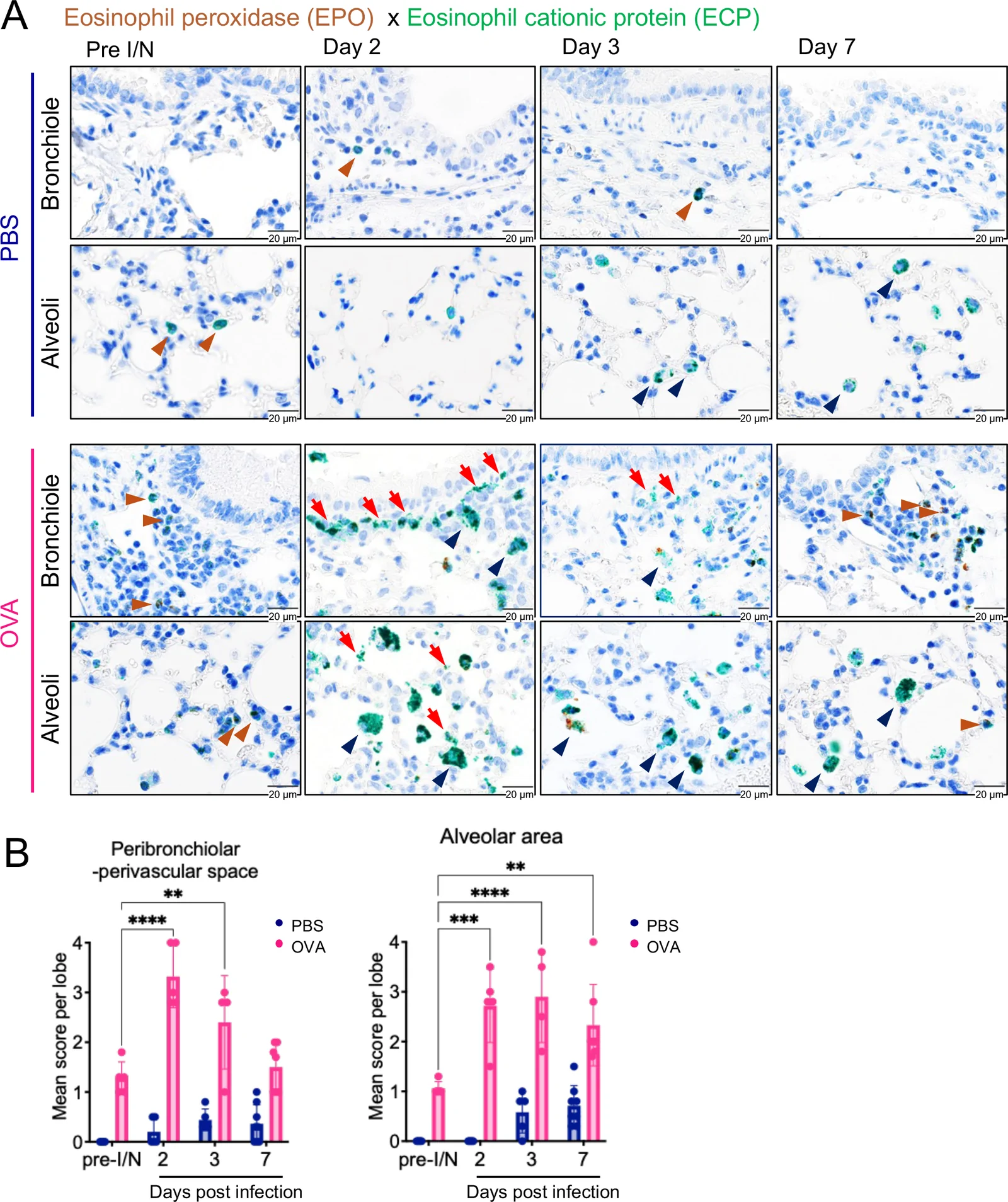
Eosinophil degranulation in the lungs of allergic mice following SARS-CoV-2 infection. (**A**) Double immunohistochemical staining for EPO (brown) and ECP (green) at pre-infection (pre I/N) and p.i. with QHmusX. Representative images of the peribronchiolar (upper panels) and alveolar (lower panels) regions are shown. Scale bars, 20 µm. Brown arrowheads indicate EPO/ECP double-positive eosinophils; red arrows indicate extracellular ECP deposition consistent with eosinophil degranulation; and black arrowheads indicate ECP-positive macrophage-like cells. (**B**) Semi-quantitative scoring of eosinophil degranulation in the peribronchiolar-perivascular space and alveolar area. Each dot represents the mean score of an individual animal. **, *P* < 0.01; ***, *P* < 0.001; and ****, *P* < 0.0001; two-way ANOVA followed by Tukey’s multiple comparisons test. Error bars indicate the mean  ±  SD.

To determine whether the rapid post-infection reduction in tissue eosinophils reflected eosinophil degranulation, we performed immunohistochemical staining for ECP and eosinophil peroxidase (EPO) (Fig. 5). ECP/EPO double staining showed few ECP/EPO double-positive eosinophils (Fig. 5A, brown arrowheads) in PBS mice throughout the observation period. Occasional ECP-positive macrophage-like cells (Fig. 5A, black arrowheads) were observed in the alveolar spaces on days 3 and 7 p.i. In OVA mice, numerous ECP/EPO double-positive eosinophils (Fig. 5A, brown arrowheads) were present before infection. By day 2 p.i., highly ECP-positive eosinophils, extracellular ECP deposition (Fig. 5A, red arrows), and ECP-positive macrophage-like cells (Fig. 5A, black arrowheads) were detected in both alveolar and peribronchiolar regions, consistent with eosinophil degranulation and phagocytic uptake of released granule proteins. Degranulation scores declined progressively in the peribronchiolar regions but remained elevated in the alveolar regions of OVA mice through day 7 p.i. (Fig. 5B). These observations support persistent eosinophil-mediated injury within the alveolar compartment following SARS-CoV-2 infection.

**Fig 6:**
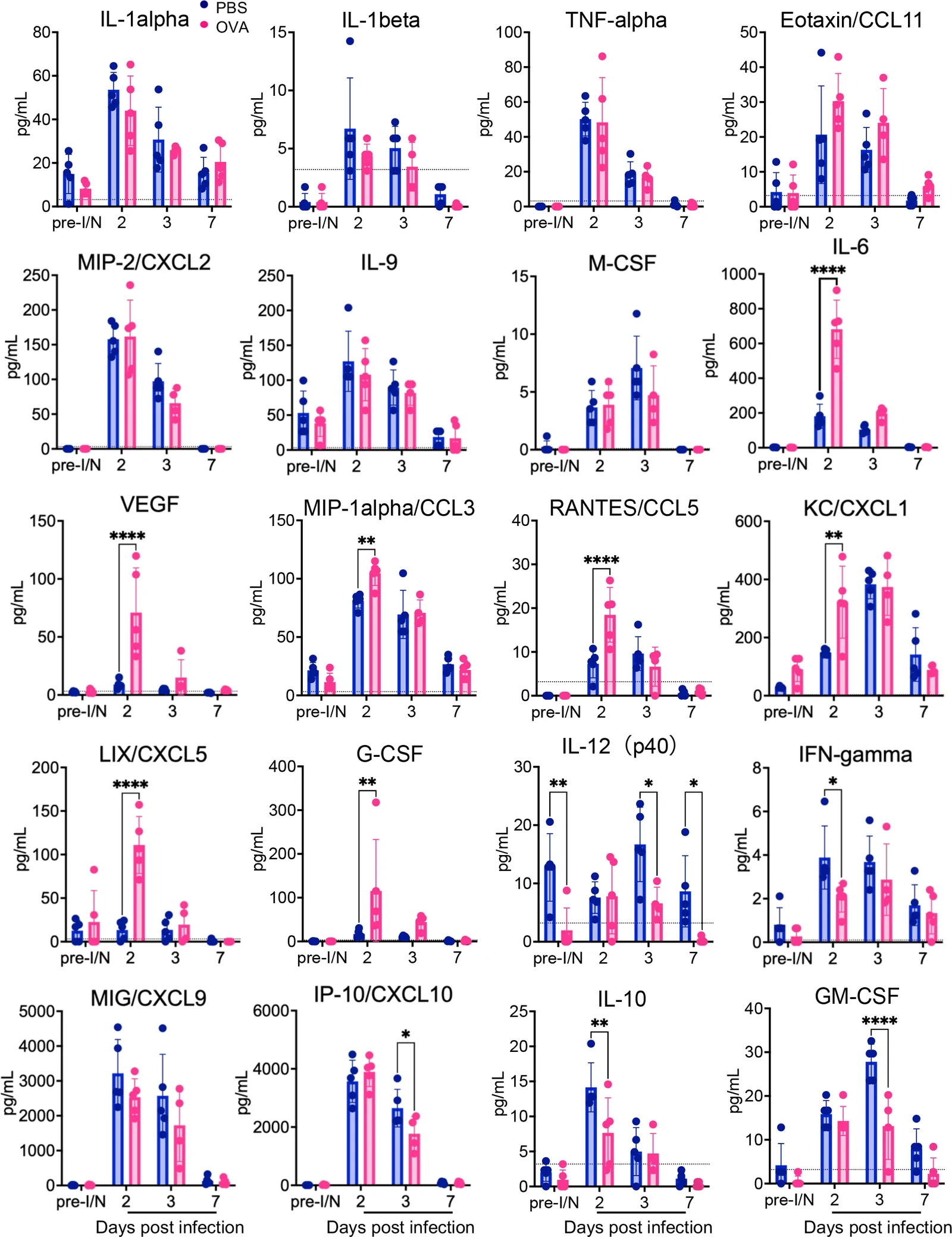
Altered cytokine and chemokine responses in the airways of allergic mice after SARS-CoV-2 infection. Cytokine and chemokine levels in BALF from the PBS and OVA groups pre-infection (pre I/N) and p.i. with QHmusX (*n* = 4 or 5 mice per group). *, *P* < 0.05; **, *P* < 0.01; and ****, *P* < 0.0001; two-way ANOVA with Šídák’s multiple comparisons test. Error bars indicate the mean  ±  SD. The pre-infection control samples were shared with our previous study (9).

Multiplex cytokine analysis of BALF revealed that IL-1α, IL-1β, tumor necrosis factor-α, eotaxin-1/C-C motif chemokine ligand 11 (CCL11), macrophage inflammatory protein (MIP)−2/C-X-C motif chemokine ligand 2 (CXCL2), IL-9, and macrophage colony-stimulating factor peaked on days 2 and 3 p.i. and returned to baseline by day 7 (Fig. 6). On day 2 p.i., OVA mice showed higher levels of IL-6, vascular endothelial growth factor (VEGF), MIP-1α/CCL3, regulated on activation, normal T-cell expressed and secreted (RANTES)/CCL5, keratinocyte-derived chemokine (KC)/CXCL1, lipopolysaccharide-induced CXC chemokine (LIX)/CXCL5, and granulocyte colony-stimulating factor (G-CSF), whereas IL-12 (p40), interferon (IFN)-γ, monokine induced by IFN-γ (MIG)/CXCL9, IFN-γ-induced protein-10/CXCL10, IL-10, and granulocyte-macrophage colony-stimulating factor were relatively lower. Among murine CXCL8 (IL-8) homologs, MIP-2/CXCL2 was induced similarly in both groups, whereas KC/CXCL1 and LIX/CXCL5 were preferentially induced in OVA mice. Notably, the representative Th2 cytokines IL-4, IL-5, and IL-13 remained below the limit of detection in both PBS and OVA mice.

RNA *in situ* hybridization followed by CD4 immunohistochemistry detected low-level *Il5* and *Il13* expression in OVA lungs before infection but not in PBS lungs (Fig. S4). After infection, weak focal *Il13* signals appeared to increase in two of five OVA mice; however, this pattern was inconsistent and less prominent than that of *Ccl11* and *Il6* expression. In contrast, *Ccl11*- and *Il6*-expressing cells were readily detected in OVA mice. *Il6*- and *Ccl11*-expressing cells were frequently localized near CD4^+^ inflammatory infiltrates but did not colocalize with CD4^+^ cells. Based on morphology, the mRNA-positive cells were consistent with mesenchymal cells, large mononuclear cells, and/or epithelial cells.

Overall, the decline in tissue eosinophils in OVA mice coincided with eosinophil degranulation, local *Il-6* and *Ccl11* expression, and enhanced granulocyte-associated chemokine induction, supporting early degranulation of pre-existing tissue eosinophils in allergic lungs following SARS-CoV-2 infection.

### Eosinophil depletion by anti-IL-5 treatment improves disease severity without affecting viral replication

**Fig 7:**
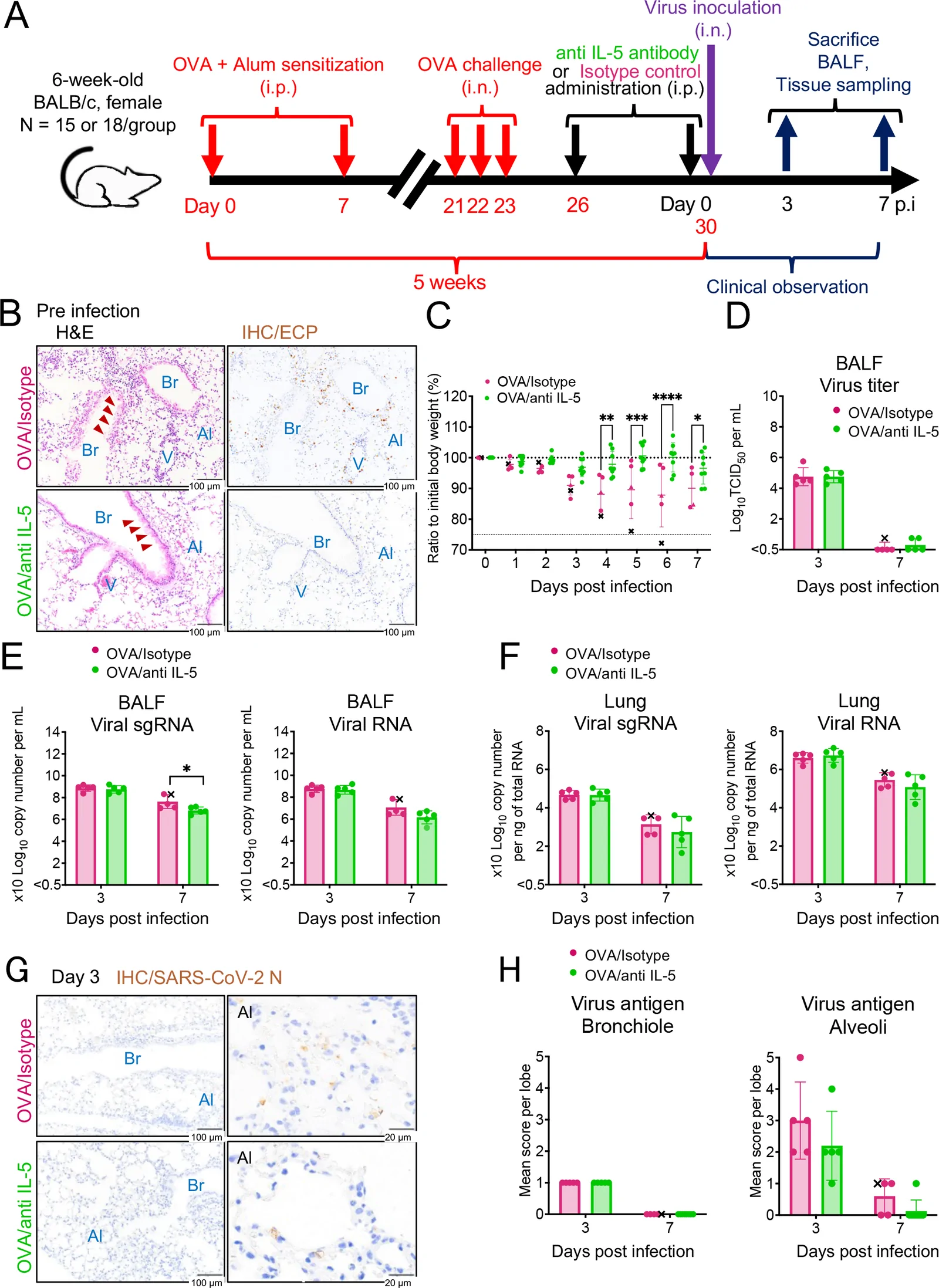
Experimental design and disease course in eosinophil-depleted OVA-induced airway allergy mice. (**A**) Schedule of OVA sensitization, challenge, and administration of anti–IL-5 monoclonal antibody in 6-week-old female BALB/c mice. Mice were aged 11 weeks at the time of infection. (**B**) Representative histopathological findings of the lungs (*n* = 5 mice per group) from the OVA/Isotype group and the OVA/anti-IL-5 group at pre-infection. IHC/ECP, immunohistochemical staining for ECP antigen. Arrowheads, mucus-secreting goblet cells in the bronchiolar epithelium; Br, bronchiole; V, vessels; Al, alveoli. (**C**) Changes in body weight after experimental infection with QHmusX. Each dot represents data from an individual animal (*n* = 5 or 8 mice per group). An animal marked with a cross indicates more than 25% weight loss and reaching a humane endpoint on day 6 (OVA/Isotype group). *, *P* < 0.05; **, *P* < 0.01; ***, *P*  <  0.001; and ****, *P* < 0.0001; two-way ANOVA followed by Šídák’s multiple comparisons test. Error bars indicate the mean  ±  SD. Bar graphs showing virus titers (**D**) and viral RNA copy number (**E**) in BALF (lower panels): sgRNA and subgenomic RNA. (**F**) Bar graph showing viral RNA copy number in the right lung lobe. *, *P* < 0.05; two-way ANOVA followed by Šídák’s multiple comparisons test (*n* = 5 mice per group). Error bars indicate the mean  ±  SD. (**G and H**) Distribution of SARS-CoV-2 antigens in the lungs of eosinophil-depleted mice after QHmusX infection. Immunohistochemistry was conducted to detect the SARS-CoV-2 N antigen. Scale bars, 20 and 100 µm. Bar graphs show scoring of viral antigen-positive cells in the bronchiole and alveoli (**H**). Error bars indicate the mean ± SD.

To directly assess the role of eosinophils, OVA mice were treated with an anti-IL-5 monoclonal antibody or an isotype control before SARS-CoV-2 inoculation (Fig. 7A). Analysis of lung tissue from the pre-infection animal group (*n* = 5) confirmed that anti-IL-5 antibody therapy following OVA sensitization significantly reduced ECP-positive eosinophils in the lungs, without affecting the presence of ACE2-negative mucus-secreting goblet cells in the bronchiolar epithelium (Fig. 7B). In OVA mice, strong ACE2-positive cells were observed focally in the peribronchiolar regions, a distribution pattern that was not altered by anti-IL-5 treatment (Fig. S5).

Following infection, all groups reached maximal weight loss on day 3 p.i.; however, anti-IL-5-treated mice began to recover their body weight by day 5 p.i. In contrast, three of the five isotype-treated mice persistently lost weight, including one that exceeded 25% body weight reduction (Fig. 7C).

Viral titers and virus-associated RNA levels in the BALF and lung tissues were comparable between anti-IL-5-treated and isotype-treated OVA mice, with no consistent differences attributable to eosinophil depletion (Fig. 7D through F). Similarly, immunohistochemical analysis showed comparable viral antigen distribution in both groups, predominantly in the alveolar regions on days 3 and 7 p.i. (Fig. 7G and H).

**Fig 8:**
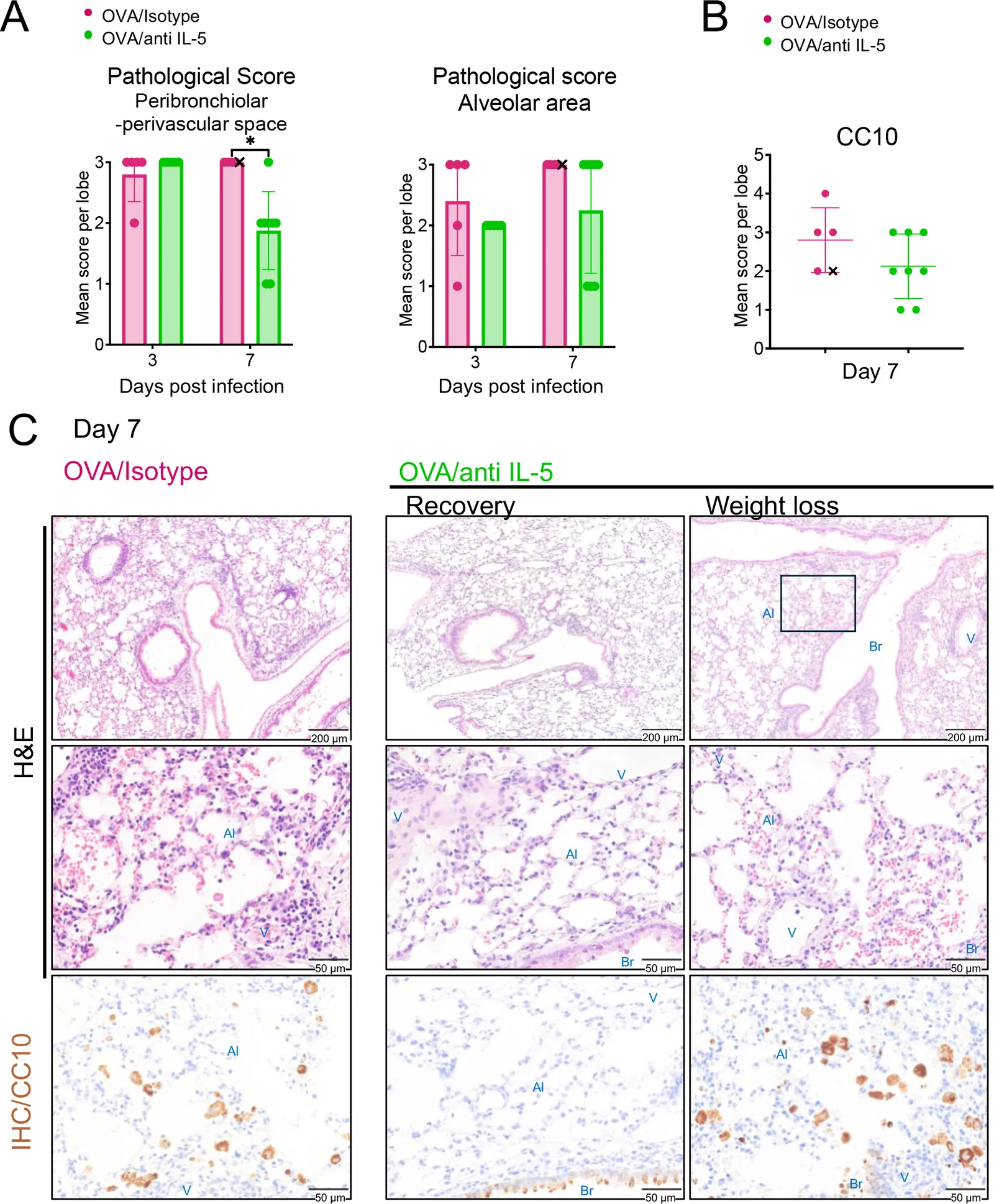
Attenuated epithelial injury and repair responses in eosinophil-depleted mice after QHmusX infection**.** (**A**) Histopathological score in the lungs of mice infected with QHmusX on days 3 and 7 p.i. (*n* = 5 or 8 mice per group). *, *P* < 0.05; Mann–Whitney test. Error bars indicate the mean ± SD. (**B**) Dot plot showing CC10 antigen scoring on day 7 p.i. Each dot represents the mean value from an individual animal. An animal marked with a cross indicates the animal that lost more than 25% body weight and reached a humane endpoint on day 6 in the OVA/Isotype group, as shown in Fig. 7C. Error bars indicate the mean  ±  SD. (**C**) Representative images showing lung pathology in the OVA/isotype and the OVA/anti-IL-5 groups on day 7 p.i. with QHmusX (*n* = 5 or 8 mice per group). Recovery: histopathology of a representative animal that recovered its body weight, as shown in Fig. 7C; weight loss, histopathology of a representative animal that continued to lose body weight, as shown in Fig. 7C. Scale bars, 200 µm and 50 µm. H&E, hematoxylin and eosin staining; CC10, immunohistochemical staining for the CC10 antigen. Br, bronchiole; V, vessels; Al, alveoli.

Histopathological analysis demonstrated that peribronchiolar–perivascular inflammatory infiltration was markedly reduced in anti-IL-5-treated OVA mice by day 7 p.i., resulting in significantly lower pathology scores than those in the isotype-treated controls (Fig. 8A). Assessment of epithelial repair revealed fewer CC10-positive cells in the alveoli of anti-IL-5-treated mice (Fig. 8B; *P* = 0.18, unpaired *t*-test). Clusters of large CC10-positive cells were observed in the alveoli of mice with mild disease but were absent in the anti-IL-5-treated OVA group mice that recovered their body weight (Fig. 8C).

**Fig 9:**
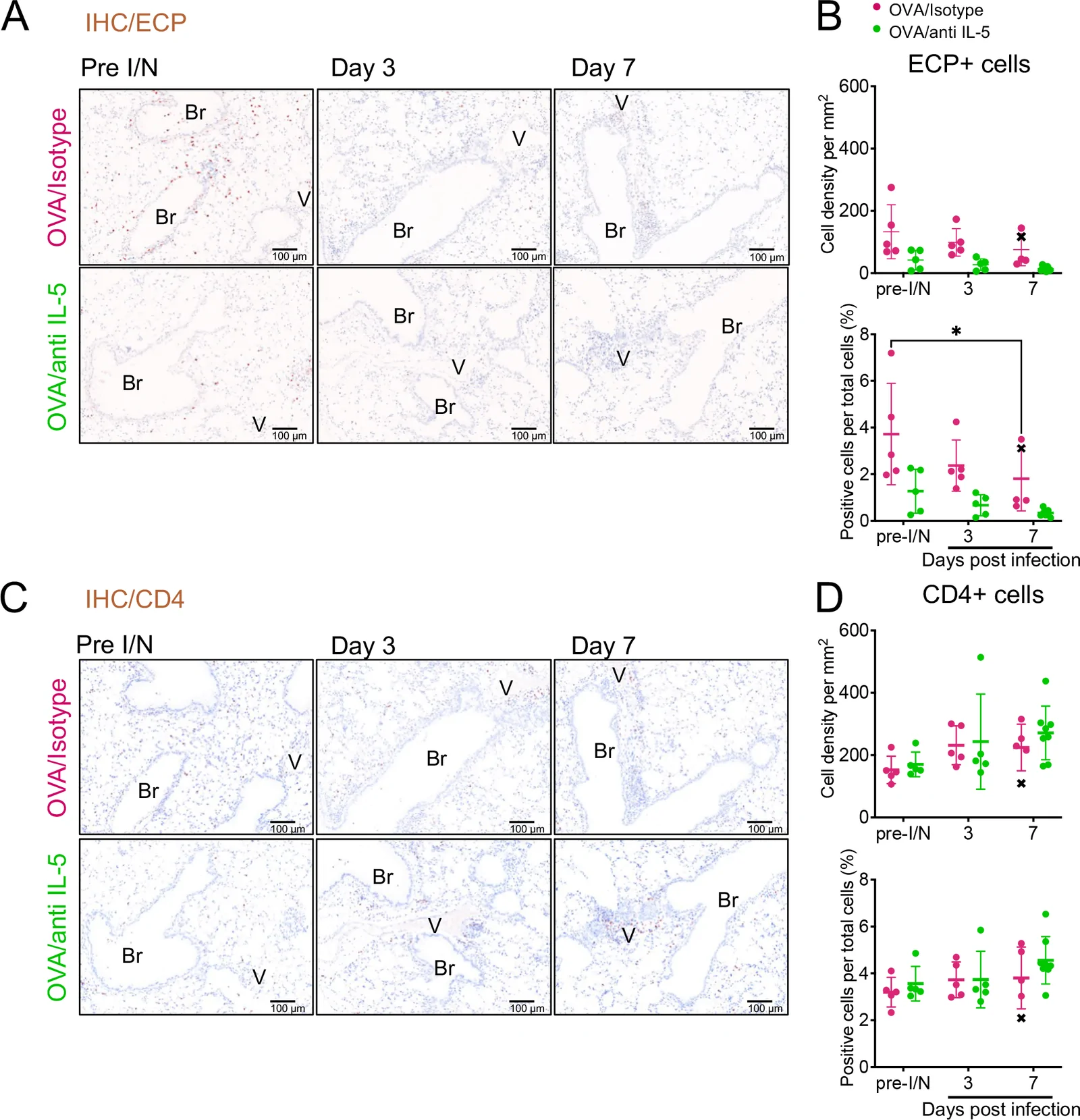
Reduced eosinophilic lung infiltration after QHmusX infection in eosinophil-depleted mice. (**A and D**) Perivascular and peribronchiolar inflammatory infiltration. Infiltration with ECP-positive eosinophils (**A and B**) and CD4^+^ T cells (**C and D**) was evaluated by digital pathology pre-infection and p.i. with QHmusX. Cells were counted using the phenotyping software inForm. Representative digital composite images are shown. The images show a region of interest within the lungs. Br, bronchiole; V, vessels. (**B and D**) Graphs showing cell density per mm^2^ and percentage positive cells. Each dot represents the mean value per lobe in an individual animal. One animal marked with a cross indicates that it lost more than 25% of its body weight and reached a humane endpoint on day 6 p.i. (OVA/Isotype group). *, *P* < 0.05; one-way ANOVA followed by Tukey’s multiple comparisons test. Error bars indicate the mean ± SD.

Anti-IL-5 treatment effectively depleted ECP-positive eosinophils in the lungs at all time points, whereas CD4^+^ T-cell dynamics were similar between the groups (Fig. 9A through D). Notably, an isotype-treated mouse with >25% body weight loss retained high levels of ECP-positive eosinophils at the humane endpoint and exhibited reduced CD4^+^ T-cell infiltration (Fig. 9B and D). The mouse showed marked bronchial epithelial remodeling but lacked large CC10-positive alveolar cell clusters (Fig. S6).

Collectively, these findings demonstrate that eosinophil depletion mitigates lung inflammation and improves clinical outcomes without affecting viral replication, supporting the pathogenic role of eosinophils in SARS-CoV-2-induced pneumonia in the context of allergic airway inflammation.

## DISCUSSION

This study demonstrates that pre-existing airway allergy substantially alters the pulmonary response to SARS-CoV-2 infection and predisposes hosts to more severe pneumonia through eosinophil-driven immunopathology. Importantly, disease severity in allergic mice was largely independent of viral load, indicating that the host immune environment, rather than viral replication itself, was the principal driver of pathology.

A defining feature of the OVA group was abundant tissue eosinophils before infection, followed by rapid eosinophil degranulation and a marked decline in tissue eosinophils after SARS-CoV-2 infection. This reduction was accompanied by extracellular ECP deposition and ECP-positive macrophage-like cells, supporting eosinophil degranulation followed by phagocytic clearance of released granule proteins. Concurrently, OVA mice exhibited enhanced induction of inflammatory and granulocyte-associated mediators in BALF, including IL-6, eotaxin-1/CCL11, KC/CXCL1, LIX/CXCL5, MIP-1α/CCL3, RANTES/CCL5, and G-CSF. Among the murine functional homologs of human CXCL8 (IL-8), MIP-2/CXCL2 was induced similarly in both groups (11), whereas KC/CXCL1 and LIX/CXCL5 were preferentially induced in OVA mice, indicating qualitative differences in the inflammatory chemokine milieu. Consistent with these observations, RNA *in situ* hybridization demonstrated abundant *Il6*- and *Ccl11*-expressing cells within inflammatory lesions, whereas *Il5* and *Il13* expression remained limited despite the Th2-biased immune background. Tryptase-positive mast cells were rarely detected in the lung parenchyma and were located mainly in peribronchial connective tissue and the pleura, suggesting that mast cells were unlikely to be major contributors to lung pathology in this model.

In contrast, early induction of the anti-inflammatory cytokine IL-10 on day 2 p.i. was attenuated in the OVA group compared with controls, suggesting impaired counter-regulatory immune responses. Together with rapid eosinophil degranulation and enhanced induction of granulocyte-associated inflammatory mediators, this inflammatory milieu coincided with increased apoptotic cell death and extensive epithelial injury, as evidenced by elevated cleaved caspase-3-positive cells during the acute phase and enhanced CC10 expression in the alveolar regions during recovery. Collectively, these findings suggest that a pre-existing Th2-dominant immune environment predisposes the lung to eosinophil-driven tissue injury, resulting in a qualitatively distinct pattern of lung damage following SARS-CoV-2 infection.

A key finding of this study is the direct contribution of eosinophils to disease exacerbation following SARS-CoV-2 infection in the OVA group. Eosinophil depletion using an anti-IL-5 antibody significantly improved weight recovery and reduced lung inflammation and pathology without affecting viral titers or viral RNA levels. These results indicate that eosinophils are dispensable for viral control in this model and instead function primarily as mediators of immunopathology. Eosinophils can have dual roles during viral infections: granule proteins, such as ECP, eosinophil-derived neurotoxin, and EPO, can contribute to antiviral host defense, whereas excessive degranulation promotes epithelial and endothelial injury, edema, and aberrant tissue remodeling (12–16). In the present study, extracellular ECP deposition and ECP-positive macrophage-like cells were observed during early infection in OVA mice, coinciding with increased apoptosis. These findings are consistent with a potential contribution of eosinophil degranulation to enhanced epithelial injury in allergic lungs. Although only slight changes in initial viral load or viral localization were observed, disease severity increased in OVA mice, suggesting that the tissue-damaging effects of eosinophilic inflammation outweighed potential antiviral benefits under allergic airway conditions. Although extracellular granule proteins were not quantified biochemically, the data support eosinophil-driven immunopathology as a major contributor to disease severity in this model.

CC10 (also known as CC16, uteroglobin, or secretoglobin-1A1) is secreted primarily by club cells, which maintain the airway epithelium and can contribute to alveolar epithelial regeneration following lung injury (17–20). In this study, large clusters of CC10-positive cells were observed within the alveoli of OVA mice with persistent weight loss after infection, likely reflecting extensive epithelial repair following severe alveolar injury. Although focal increases in CC10-positive cells were also observed in infected PBS mice, OVA mice exhibited markedly greater and more widespread accumulation, suggesting an exaggerated epithelial repair/remodeling response associated with allergic airway inflammation. In contrast, these CC10-positive clusters were absent in anti-IL-5-treated OVA mice that recovered body weight, consistent with reduced epithelial injury and diminished regenerative demand. Interestingly, large CC10-positive clusters were also absent in a severely affected outlier mouse with >25% weight loss on day 6 p.i., suggesting that extremely severe injury may impair effective repair, possibly through progenitor cell depletion or dysfunction. Although speculative, this observation raises the possibility that severe virus-induced lung damage disrupts regenerative capacity.

Importantly, allergic airway inflammation also reshapes the spatial distribution of SARS-CoV-2-susceptible cells. In our previous study using the same OVA-induced allergy model, ACE2-expressing cells were reduced in the bronchiolar epithelium and enriched in the alveolar regions (9). This altered cellular landscape likely promotes localization of infection to alveoli, thereby amplifying tissue injury and inflammatory responses, including eosinophil-mediated immunopathology, even when viral replication is limited.

Consistent with our previous findings, OVA mice developed a Th2-biased humoral immune response following SARS-CoV-2 infection, as reflected by increased spike- and RBD-specific IgG1/IgG2a ratios (9). Notably, this Th2-biased response was selective for spike and RBD, whereas N-specific IgG1 and IgG2a responses were comparable between groups. These findings suggest that pre-existing allergic immune polarization may influence humoral responses in an antigen-dependent manner. However, the mechanisms underlying this antigen-specific difference remain to be elucidated. Despite these differences in antigen-specific IgG subclass responses, neutralizing antibody titers were comparable between the two groups, indicating that impaired neutralizing antibody responses do not explain the enhanced disease severity observed in OVA mice.

These findings help contextualize heterogeneous clinical observations in patients with asthma and airway allergies. Epidemiological studies have reported neutral (3, 4), detrimental (5–7), and protective (8) associations between asthma and COVID-19 severity, likely reflecting differences in asthma endotypes, inflammatory states at infection, treatment regimens, and individual immune responses. Our animal model suggests that, during active allergic inflammation, eosinophils amplify lung injury during SARS-CoV-2 infection and contribute to severe pneumonia independently of viral load.

The results also have therapeutic implications. IL-5-targeted biologics are widely used for eosinophilic asthma (21–23), and eosinophil depletion improved disease severity in this model. Although eosinophils may contribute to antiviral defense in certain contexts, our findings indicate that targeted suppression of eosinophil-driven inflammation can be beneficial during acute SARS-CoV-2-induced pneumonia in allergic hosts. However, the timing and clinical context of such interventions require careful consideration. Future studies should evaluate long-term outcomes, including lung remodeling, fibrosis, and progenitor cell function, to clarify how allergic inflammation influences chronic sequelae following SARS-CoV-2 infection.

Several limitations should be acknowledged. First, the OVA group represents an acute and relatively homogeneous Th2-skewed immune environment (9) and does not fully recapitulate the clinical heterogeneity of human asthma, including chronic airway remodeling or non-Th2 phenotypes. Second, although Th2-biased immune responses were evaluated using antigen-specific IgG subclasses, cytokine profiling, RNA *in situ* hybridization, and CD4 immunohistochemistry, Th2 cells were not directly characterized by flow cytometry or intracellular cytokine staining. Third, although eosinophil degranulation was supported histopathologically by ECP/EPO staining, extracellular ECP deposition, and ECP-positive macrophage-like cells, extracellular granule proteins were not quantified biochemically, and the molecular mechanisms regulating eosinophil activation remain to be elucidated. Fourth, although the mouse-passaged QHmusX strain enables robust pneumonia modeling, it may differ in tissue tropism and immunogenicity from currently circulating Omicron variants. Fifth, although eosinophil depletion implicates eosinophils as key drivers of pathology, contributions from other Th2-associated pathways cannot be excluded completely (24). In addition, the potential contribution of complement activation and complement-derived anaphylatoxins to eosinophil recruitment, activation, or degranulation was not evaluated. Although only limited and inconsistent *Il13* expression was detected during the early phase of infection, potential contributions of IL-13 to later epithelial remodeling or mucus production remain possible. Finally, although increased CC10 expression suggested activation of epithelial repair responses, the long-term consequences of eosinophil-mediated injury, including aberrant epithelial repair, progenitor cell dysfunction, and fibrotic remodeling, were not addressed and warrant further investigation.

### Conclusion

This study demonstrates that preexisting allergic airway inflammation alters the pulmonary response to SARS-CoV-2 infection and exacerbates pneumonia through rapid eosinophil activation and degranulation. Early eosinophil degranulation, together with the protective effect of anti-IL-5 treatment in OVA mice, supports a key role for eosinophils in disease progression. These findings provide new insights into how a pre-existing allergic immune environment shapes the host response to SARS-CoV-2 infection and contributes to severe viral pneumonia.

## MATERIALS AND METHODS

### Viruses and cells

The mouse-passaged SARS-CoV-2 strain QHmusX was derived from the human isolate hCoV-19/Japan/QH-329-037/2020 after 10 passages in BALB/c mice, followed by two passages in VeroE6/TMPRSS2 cells (10). VeroE6/TMPRSS2 cells (JCRB1819) were maintained in DMEM containing 10% FBS and antibiotics and were used for viral propagation and titration (25). Infectious titers were determined by the TCID₅₀ method using the Behrens–Kärber calculation. All procedures involving SARS-CoV-2 were conducted in a Biosafety Level 3 (BSL-3) laboratory.

### Animals

Female BALB/c mice (6 weeks old; Japan SLC) were housed under SPF conditions. The humane endpoint was defined as the appearance of clinical signs of respiratory stress, including respiratory distress and >25% weight loss. The animals were euthanized under anesthesia using an overdose of isoflurane if severe disease symptoms or weight loss were observed.

### OVA-induced allergic airway inflammation

OVA-induced airway inflammation was established as described previously, with minor modifications (9, 26, 27). Briefly, mice were sensitized twice by intraperitoneal injection of 100 μg OVA with 2 mg of alum at 1-week intervals and challenged intranasally with 100 μg OVA on three consecutive days (days 21–23) under anesthesia. The control mice were administered PBS instead of OVA. Serum OVA-specific IgE levels were measured using an LBIS Mouse anti-OVA-IgE ELISA Kit (Fujifilm Wako Chemicals Co.) 1 week before SARS-CoV-2 infection. The present study was conducted simultaneously with our previous study using the same experimental cohort and identical sensitization, infection, and sampling protocols (9). Therefore, pre-infection control animals common to both studies were shared for analyses performed before infection to minimize animal use in accordance with the 3Rs principle. A schematic of the sensitization and challenge protocols is shown in Fig. 1A.

### SARS-CoV-2 infection of allergic mice

Five weeks after the first sensitization, mice were transferred to the BSL3 facility and infected intranasally with 3 × 10³ TCID₅₀ of QHmusX under ketamine–xylazine anesthesia. This viral dose was sufficient to cause transient weight loss due to acute pneumonia in 11-week-old BALB/c mice without lethality. Body weight was monitored daily for 7 days p.i. To assess virological and pathological outcomes, subsets of mice were euthanized under anesthesia with an overdose of isoflurane on days 2, 3, and 7 p.i. to collect BALF and lung tissues. Extrapulmonary organs were not systematically examined in this study. Humane endpoints included >25% weight loss and severe respiratory distress.

### Eosinophil depletion using anti-IL-5 antibody

OVA group received intranasal administration of 0.05 mg anti-IL-5 monoclonal antibody (InVivoMAb anti-mouse/human IL-5, TRFK5, Bio X Cell, Lebanon, NH) or rat IgG1 isotype control (Bio X Cell) on days 26 and 30 after initial sensitization. Eosinophil depletion was confirmed in the lung tissue. The mice were subsequently infected with QHmusX. The detailed schedule is shown in Fig. 6A.

### Collection of BALF

Under anesthesia with an overdose of isoflurane, the murine trachea was cannulated, and the lungs were washed with 1 mL PBS containing 0.1% BSA and antibiotics to obtain BALF (9). Samples were clarified by centrifugation and stored at −80°C.

### Measurement of SARS-CoV-2-specific IgG subclasses

SARS-CoV-2 spike-, RBD-, and N-specific IgG1 and IgG2a antibodies were measured using the MSD Coronavirus Plate 3 Multi-Spot assay in combination with SULFO-TAG-conjugated anti-mouse IgG1 and IgG2a detection antibodies from the MSD Mouse Isotyping Panel 1 kit (Meso Scale Diagnostics, Rockville, MD, USA), according to the manufacturer’s instructions.

### RNA extraction and viral RNA quantification

RNA was extracted from BALF and right lower lung lobe tissues using Maxwell RSC kits (Promega). Viral genomic RNA and subgenomic N transcripts were quantified using RT-qPCR (LightCycler 480) with published primer–probe sets (Table S1) (9, 28). Reaction conditions were 50°C for 30 min, 95°C for 15 min, followed by 40 cycles of 94°C for 15 s and 60°C for 60 s on a LightCycler 480 system (Roche, Basel, Switzerland).

### Histopathology and immunohistochemistry

Lungs were fixed in 10% formalin, embedded in paraffin, sectioned, and stained with H&E. SARS-CoV-2 N antigen and host markers (cleaved caspase-3, ECP, CD4, MPO, EPO, tryptase, CC10, and ACE2; Table S2) were detected through immunohistochemistry using standard antigen retrieval and polymer-based detection systems (Nichirei Biosciences, Inc., Tokyo, Japan; Leica Biosystems, Germany) and an automated immunostaining system (Leica Bond RX^m^; Leica Biosystems) (9). Sequential double immunohistochemical staining was performed for cleaved caspase-3 and SARS-CoV-2 antigen, EPO and ECP, ECP and MPO, and mast cell tryptase and ECP, with heat-mediated antibody denaturation between the staining steps.

Images for ECP and CD4 were acquired using a PhenoImager Fusion system (Akoya Biosciences, Marlborough, MA, USA), and digital quantification of eosinophils and CD4^+^ T cells was performed using inForm software (Akoya Biosciences).

For chromogenic ECP/MPO double immunohistochemistry, whole-slide images of double-stained left lung sections were acquired using a VS200 digital slide scanner (Evident, Tokyo, Japan) and analyzed using QuPath (version 0.7.0). ECP-positive eosinophils and MPO-positive neutrophils were quantified separately in the peribronchiolar and alveolar regions of scanned left lung sections.

### RNA *in situ* hybridization

RNA *in situ* hybridization was performed on formalin-fixed, paraffin-embedded lung sections using the RNAscope platform (Advanced Cell Diagnostics, Newark, CA, USA) on a BOND RX^m^ automated staining system (Leica Biosystems) according to the manufacturer’s instructions. The probes used in this study are listed in Table S3. Sections were subsequently subjected to CD4 immunohistochemistry.

### Pathology scoring

Peribronchiolar/perivascular and alveolar inflammation were semi-quantitatively scored on a 0–3 scale (0, none; 1, mild; 2, moderate; and 3, severe) according to previously described criteria (9, 29).

Viral antigen and cleaved caspase-3 immunoreactivity were independently evaluated using semi-quantitative scoring systems (0–3 or 0–4 scales) based on the number and distribution of positively stained cells, as previously described (9, 29).

CC10 immunoreactivity was assessed using a separate semi-quantitative scoring system based on the number of CC10-positive cells per tissue section as follows: 0, no positive cells; 1, 1–25 positive cells; 2, 26–50 positive cells; 3, 51–75 positive cells; 4, 76–100 positive cells; and 5, >100 positive cells per section.

Eosinophil degranulation was semi-quantitatively scored on ECP/EPO double-immunostained lung sections using a five-point scale (0–4) as follows: 0, the normal range defined using PBS-treated mice before infection; 1, diffuse distribution of EPO^+^/ECP^+^ eosinophils within a single lung lobe and/or fewer than three weakly ECP-positive macrophage-like cells per field using a 20× objective; 2, 3–5 strongly ECP-positive macrophage-like cells in one field; 3, 3–5 strongly ECP-positive macrophage-like cells in two or more fields; and 4, ≥6 strongly ECP-positive macrophage-like cells in one field within a single lung lobe.

### Cytokine and chemokine measurement

Cytokine and chemokine levels in BALF were quantified using a 32-plex mouse magnetic bead panel (Millipore). The samples were UV-inactivated and analyzed using a Luminex 200 system according to the manufacturer’s instructions, as previously described (9, 10).

### Statistical analysis

All data are expressed as the mean ± SD, except for data pertaining to OVA-specific IgE, IgG, and neutralizing antibodies (expressed as GMT with 95% confidence intervals, GMT + 95% CI). Data distribution was assessed using the Shapiro–Wilk test, and parametric or nonparametric statistical analyses were selected accordingly. Statistical tests (ANOVA or nonparametric equivalents) were performed using GraphPad Prism 10 and 11, with *P* <0.05 considered statistically significant.

## Data Availability

The full genome sequence of the mouse-passaged SARS-CoV-2 QHmusX strain used in this study has been deposited in the DNA Data Bank of Japan under accession no. LC605054. All other study data are included in the article and/or supplemental material.

## SUPPLEMENTAL MATERIAL

Supplemental material msphere.00123-26-s0001.pdf Tables S1 to S3; Fig. S1 to S6.

ASM does not own the copyrights to Supplemental Material that may be linked to, or accessed through, an article. The authors have granted ASM a non-exclusive, world-wide license to publish the Supplemental Material files. Please contact the corresponding author directly for reuse.
