# Supplementary material for: Eosinophil-driven immunopathology exacerbates SARS-CoV-2 pneumonia in an OVA-induced airway allergy mouse model
Source: mSphere. 2026 Sep 28;11(10):e00123-26. doi: 10.1128/msphere.00123-26 (PMC13711792; doi:10.1128/msphere.00123-26)
Supplement: Supplementary file 1 [file msphere.00123-26-s0001.pdf]

Table S1

Table S1. Primer and probe sets used in the study

Target	Primer/probe	Sequence 5' ->3'
**SARS-CoV-2 N gene**	NIID_2019-nCOV_N_F2	5'-AAATTTTGGGGACCAGGAAC-3'
	NIID_2019-nCOV_N_R2	5'-TGGCAGCTGTGTAGGTCAAC-3'
	NIID_2019-nCOV_N_P2	5'-FAM-ATGTCGCGCATTGGCATGGA-BHQ-3'
**SARS-CoV-2 subgenomic**	SARS2-LeaderF60	5'-CGATCTCTTGTAGATCTGTTCTCT-3'
	SARS2-N28354R	5'-TCTGAGGGTCCACCAAACGT-3'
	SARS2-N28313Fam	5'-FAM-TCAGCGAAATGCACCCCGCA-TAMRA-3'

Table S2

Table S2. Primary antibodies used for immunohistochemistry

Target	Antibody (clone)	Catalog no. /manufacturer	Antigen retrieval	Application
**SARS-CoV-2 N**	Rabbit anti-SARS-CoV-2 Nucleocapsid protein	In house	pH6*, ** 95°C	Viral antigen
**Cleaved Caspase-3**	Cleaved Caspase-3 (Asp175) Antibody	#9661/ Cell Signaling Technology (CST, Danvers, MA)	pH9*** 95°C	Apoptosis detection
**CC10/ uteroglobin**	Anti-Uteroglobin antibody (EPR19846)	ab213203 /Abcam (Cambridge, UK)	pH6** 95°C	CC10 expression
**ECP (for single staining)**	Rabbit Anti-Mouse Eosinophil Cationic Protein (ECP)	A00128-03-100 /Aviscera Bioscience (Santa Clara, CA)	Proteinase K (RE7160-K, Leica), 37°C	Eosinophil detection
**CD4**	Anti-CD4 antibody (EPR19514)	ab183685 /Abcam	pH6**, 95°C	T-cell detection
**ECP (for double staining)**	Eosinophil cationic protein (F3G7N) Rabbit Monoclonal Antibody	#99106 /CST	pH6**, 95 or 100°C****	Eosinophil detection/degranulation
**Myeloperoxidase (MPO)**	MPO Polyclonal antibody	22225-1-AP /Proteintech (Chicago, IL)	pH9***, 100°C	Neutrophil detection
**Tryptase**	Tryptase (E7M2U) Rabbit Monoclonal Antibody	#19523 /CST	pH6* 95°C	Mast cell detection
**Eosinophil peroxidase (EPO)**	Eosinophil peroxidase (F3C2H) Rabbit Monoclonal Antibody	#98757 /CST	pH6**, 100°C	Eosinophil detection
**ACE2**	ACE2 Recombinant (SN0754) Rabbit Monoclonal Antibody	MA5-32307 /Invitrogen	pH6**	ACE2 expression

*Deparaffinization/Antigen Retrieval Solution, pH6 (415281; Nichirei Biosciences, Tokyo, Japan),

**BOND-PRIME Epitope Retrieval Solution 1 (AR0086; Leica Biosystems, Nussloch Germany)

***BOND-PRIME Epitope Retrieval Solution 2 (AR0087; Leica Biosystems))

**** 95°C or 100°C, depending on the staining protocol

Table S3

Table S3. Probes for RNA *in situ* hybridization used in the study

Target gene	Probe name	Catalog no.	Purpose
Il5	RNAscope LS 2.5 Probe-Mm-Il5	319388	Th2-associated cytokine
Il13	RNAscope LS 2.5 Probe-Mm-Il13	312298	Th2-associated cytokine
Ccl11	RNAscope LS 2.5 Probe-Mm-Ccl11	464038	Eosinophil-associated chemokine
Il6	RNAscope LS 2.5 Probe-Mm-Il6	315898	Inflammatory cytokine

Fig S1

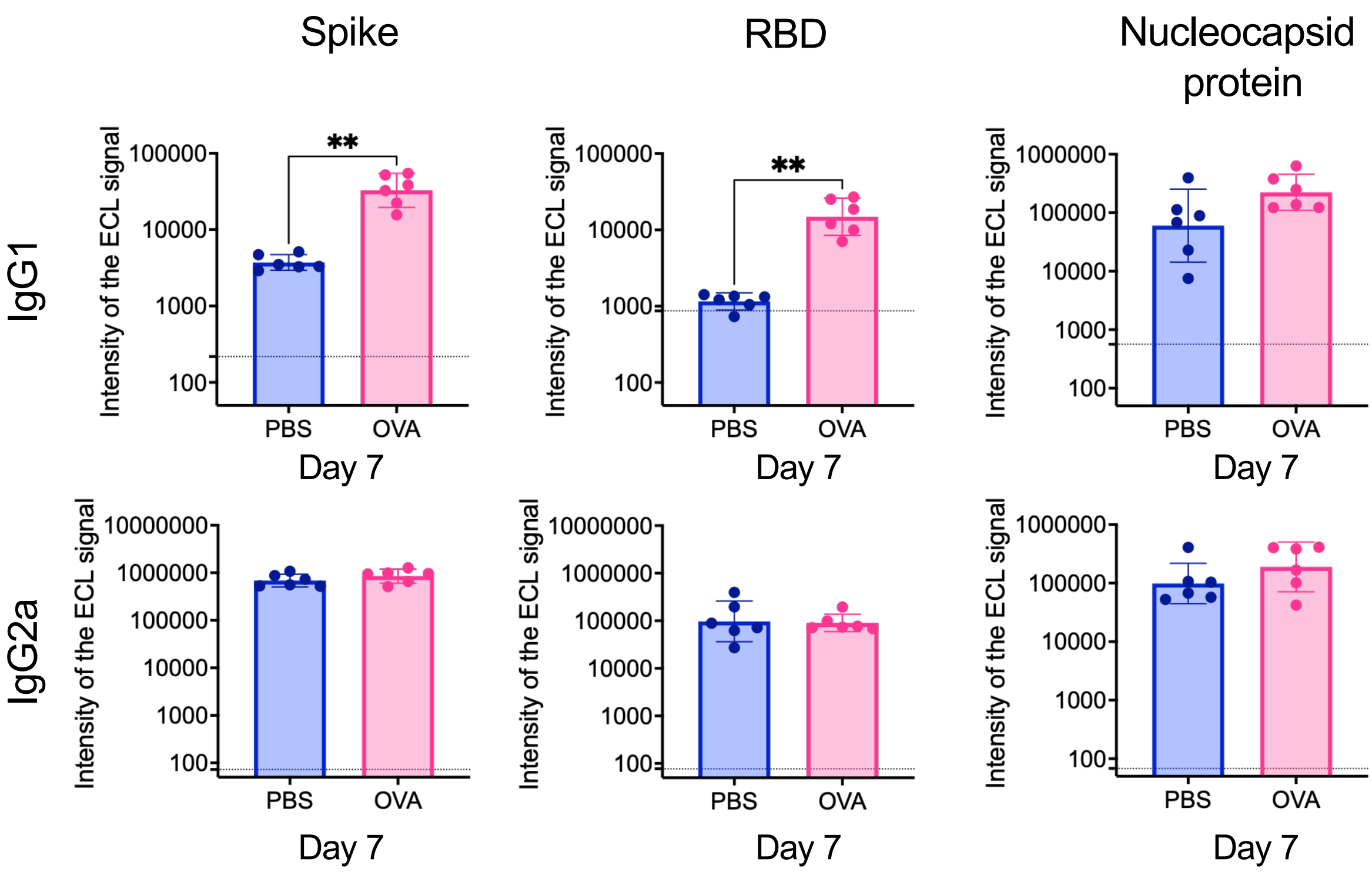

Fig. S1 SARS-CoV-2 antigen-specific IgG1 and IgG2a responses in PBS and OVA mice at day 7 post-infection. Serum spike-, receptor-binding domain (RBD)-, and nucleocapsid protein-specific IgG1 and IgG2a responses were measured on day 7 post-infection using the MSD Coronavirus Plate 3 Multi-Spot assay. Serum samples were diluted 1:200, and antibody responses are presented as electrochemiluminescence (ECL) signal intensity. Each dot represents one mouse. The dashed line indicates the mean value of four individual pre-infection samples (two PBS and two OVA mice).**P < 0.01; unpaired two-tailed t-test. Error bars indicate the GMT with 95% CI.

Fig S2

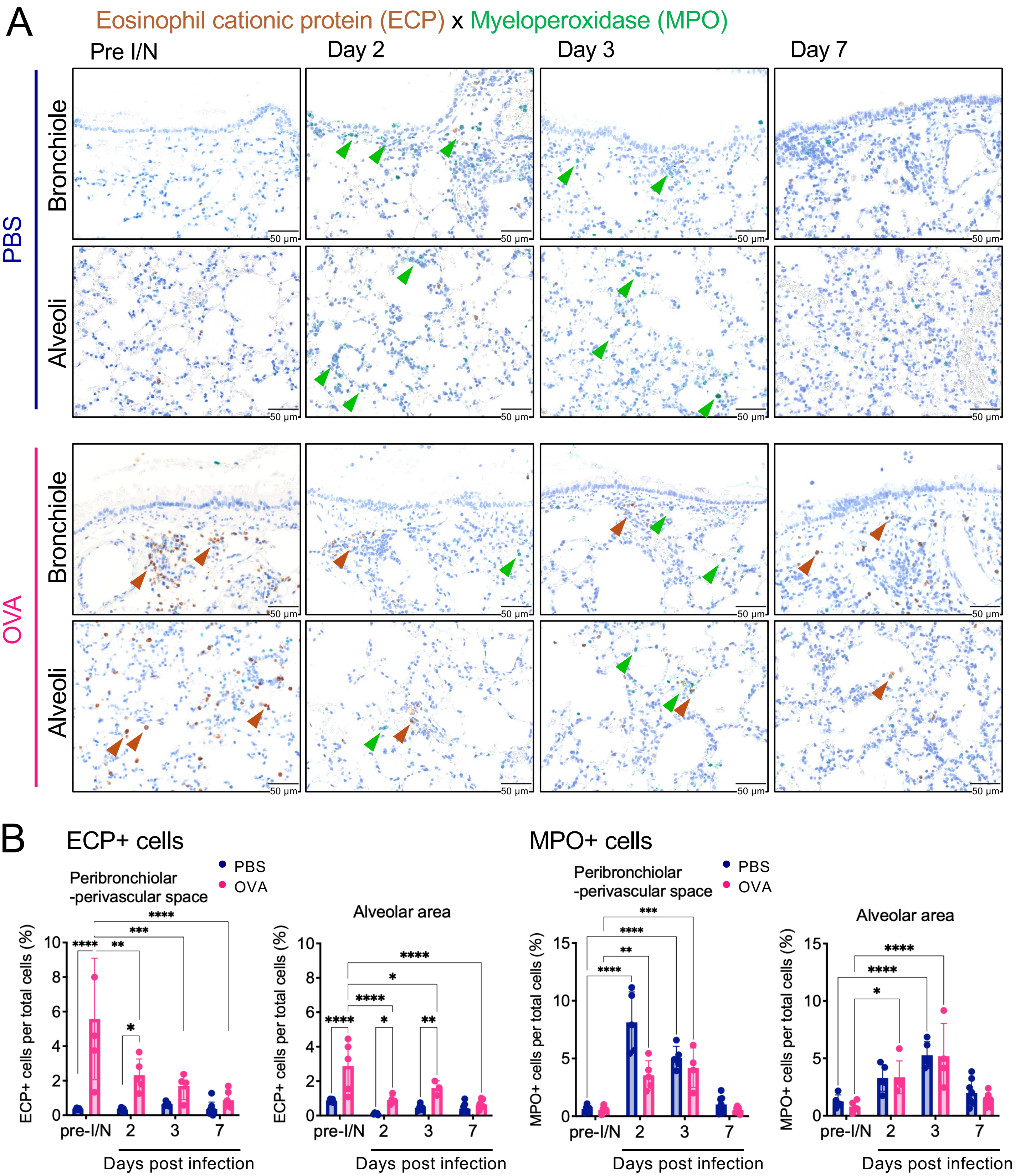
A
Eosinophil cationic protein (ECP) x Myeloperoxidase (MPO)
Pre I/N
Day 2
Day 3
Day 7
PBS
OVA
Bronchiole
Alveoli
50 μm
B
ECP+ cells
MPO+ cells
Peribronchiolar
-perivascular space
Alveolar area
PBS
OVA
ECP+ cells per total cells (%)
MPO+ cells per total cells (%)
pre-I/N
2
3
7
Days post infection

Fig. S2 Eosinophil and neutrophil infiltration in the lungs of QHmusX-infected mice. (A) Double immunohistochemical staining for eosinophilic cationic protein (ECP; brown) and myeloperoxidase (MPO; green), identifying eosinophils and neutrophils, respectively. Representative images of the peribronchiolar (upper panels) and alveolar (lower panels) regions are shown. Arrowheads indicate eosinophil accumulation (brown) and neutrophil accumulation (green). Scale bars, 50 μm. (B) Quantitative analysis of ECP-positive cells and MPO-positive cells in the lungs pre-infection (pre I/N) and post-infection (p.i.) with QHmusX. Cells were quantified separately in the annotated peribronchiolar-perivascular space and alveolar area of the left lung using the digital pathology software QuPath (0.7.0-arm64). Each dot represents an individual animal. *, $P < 0.05$, **, $P < 0.01$, ***, $P < 0.001$, ****, $P < 0.0001$; two-way ANOVA followed by Tukey's multiple comparisons test. Error bars indicate the mean ± SD.

Fig S3

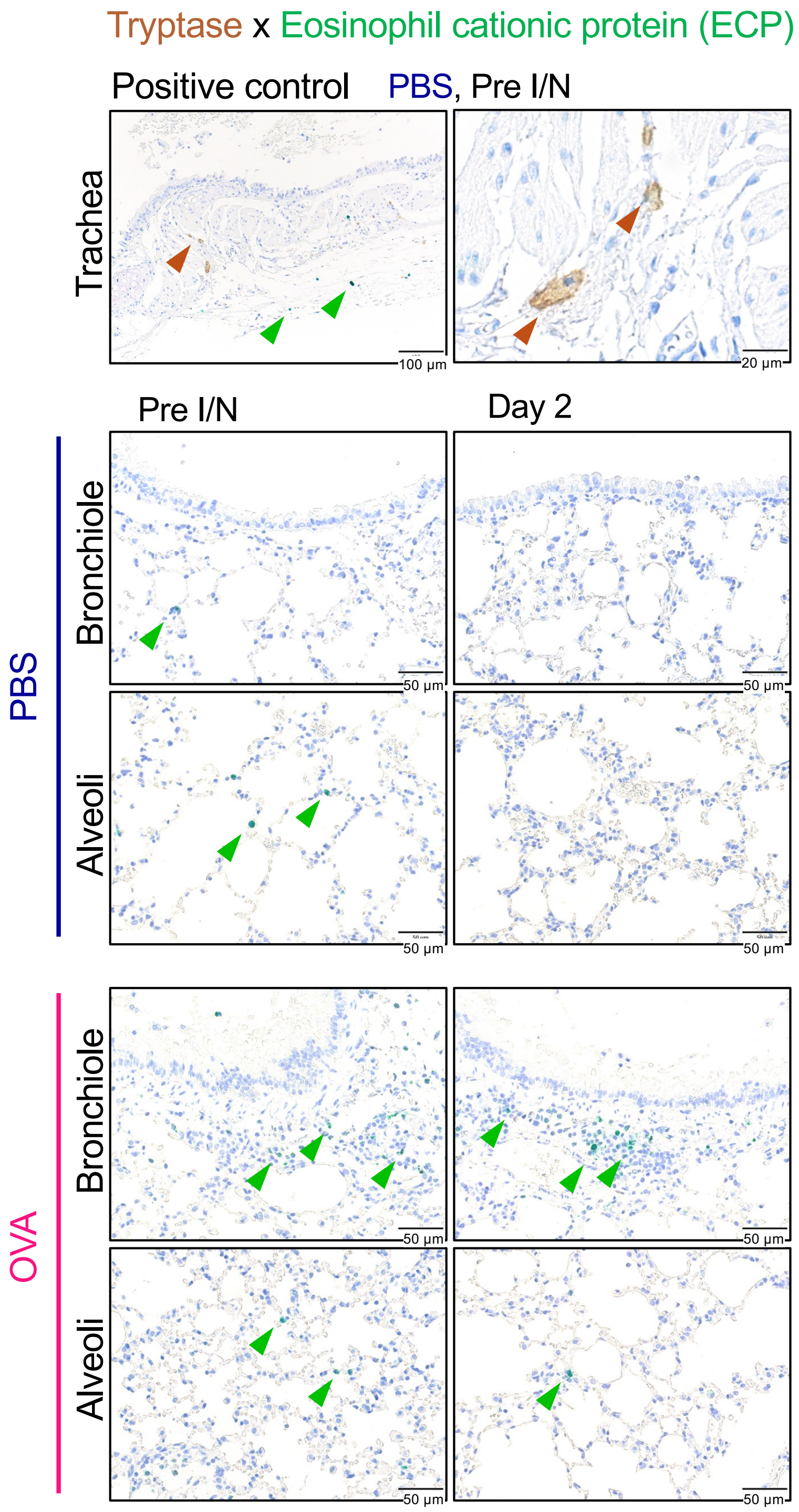

Fig. S3 Limited detection of tryptase-positive mast cells in the lungs of QHmusX-infected PBS and OVA mice. Double immunohistochemical staining for mast cell tryptase (brown) and eosinophil cationic protein (ECP; green) in lung sections from PBS- and OVA-treated mice pre-infection (pre I/N) and at day 2 post-infection. A tracheal section from a PBS-treated mouse, obtained before infection, was used as a positive control for tryptase staining. Tryptase-positive mast cells (brown arrowheads) are shown in the positive-control and peribronchial connective-tissue images and were rarely observed in the lung parenchyma. Green arrowheads indicate ECP-positive eosinophils. Representative images are shown. Scale bars, 100, 20, and 50 µm.

Fig S4

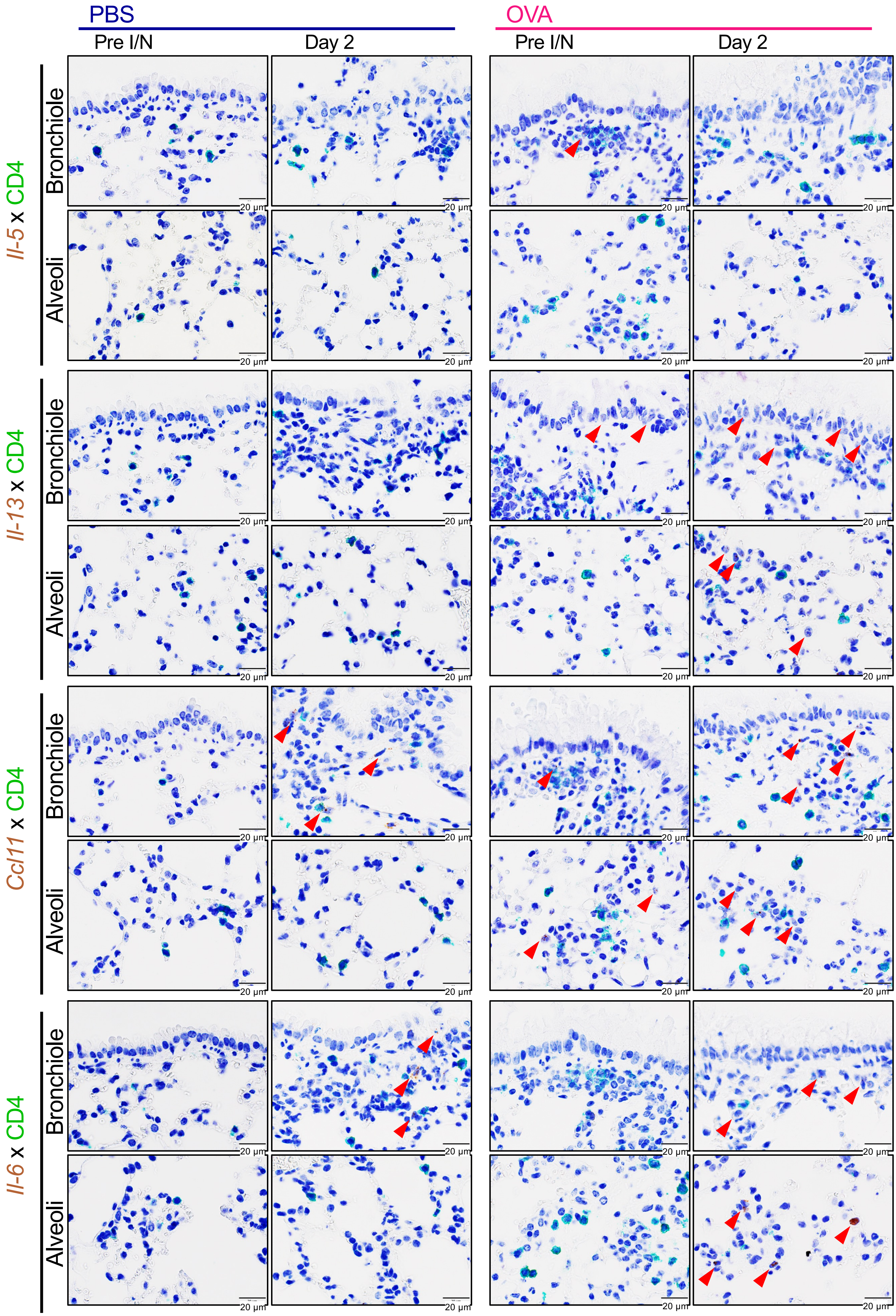

PBS
OVA
Pre I/N
Day 2
Pre I/N
Day 2
Il-5 x CD4
Il-13 x CD4
Ccl11 x CD4
Il-6 x CD4
Bronchiole
Alveoli
20 μm

Fig. S4 Tissue localization of *Il5*, *Il13*, *Ccl11*, and *Il6* mRNA in the lungs of QHmusX-infected mice. RNA *in situ* hybridization was performed to detect *Il5*, *Il13*, *Ccl11*, and *Il6* mRNA (brown dots), followed by immunohistochemistry for CD4 (green). Representative images of the peribronchiolar (upper panels) and alveolar (lower panels) regions are shown (n = 4 or 5 mice per group). Red arrowheads indicate mRNA-positive cells. Scale bars, 20 μm.

Fig S5

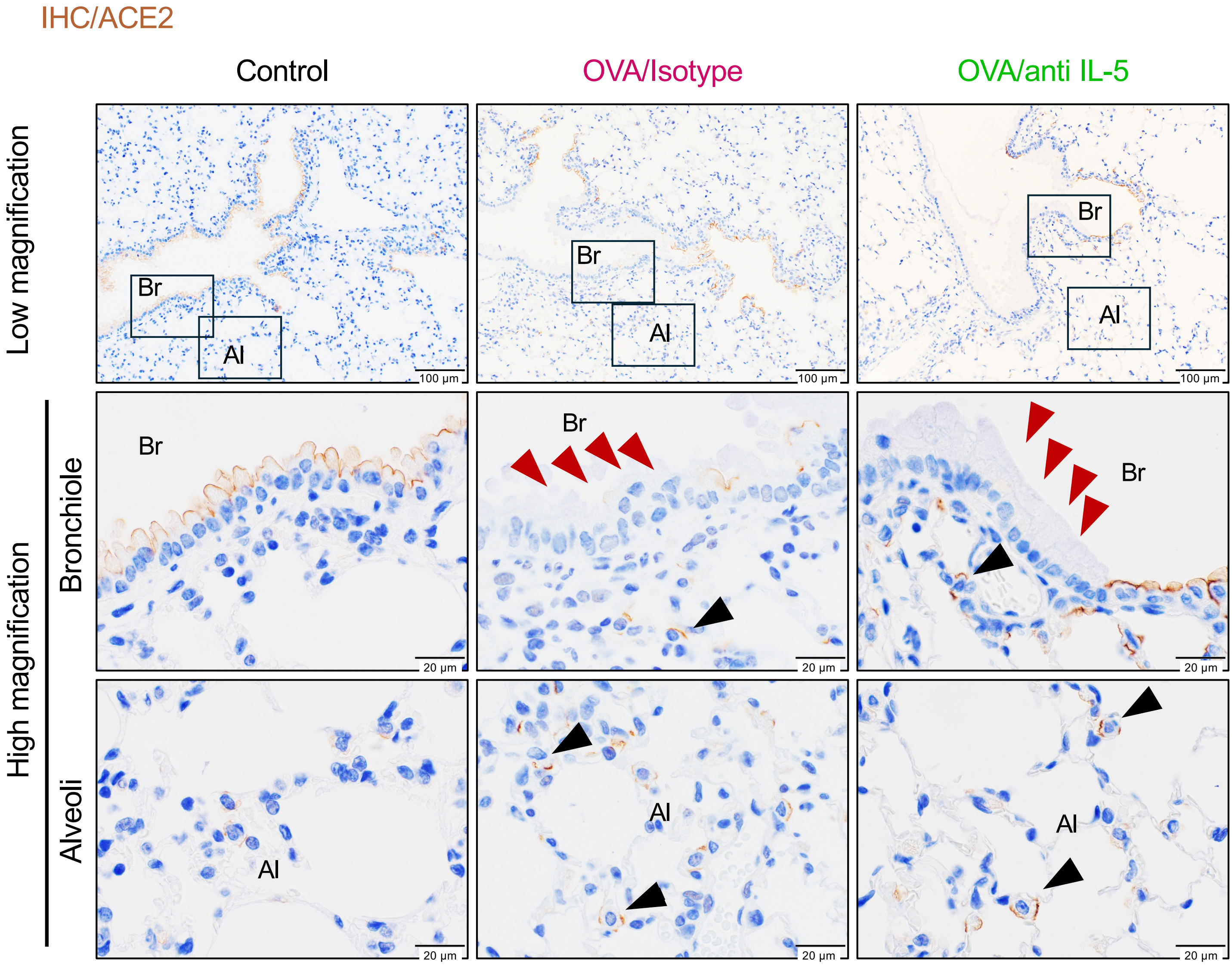

Fig. S5 ACE2 expression patterns in the bronchiolar and alveolar regions of OVA-induced airway allergy mice. Representative histopathological findings of the lungs (n = 5 mice per group) from non-OVA-sensitized control mice, OVA/Isotype group, and OVA/anti–IL-5 group at pre-infection. IHC/ACE2, immunohistochemical staining for ACE2 antigen. Mucus-secreting goblet cells in the bronchiolar epithelium were negative for ACE2 staining (red arrowheads). Strongly ACE2-positive cells were observed focally in peribronchiolar and alveolar regions of OVA-treated mice (black arrowheads). The ACE2 staining pattern was not apparently altered by anti–IL-5 treatment. Br, bronchiole; Al, alveoli. Scale bars, 100 μm and 20 μm.

Fig S6

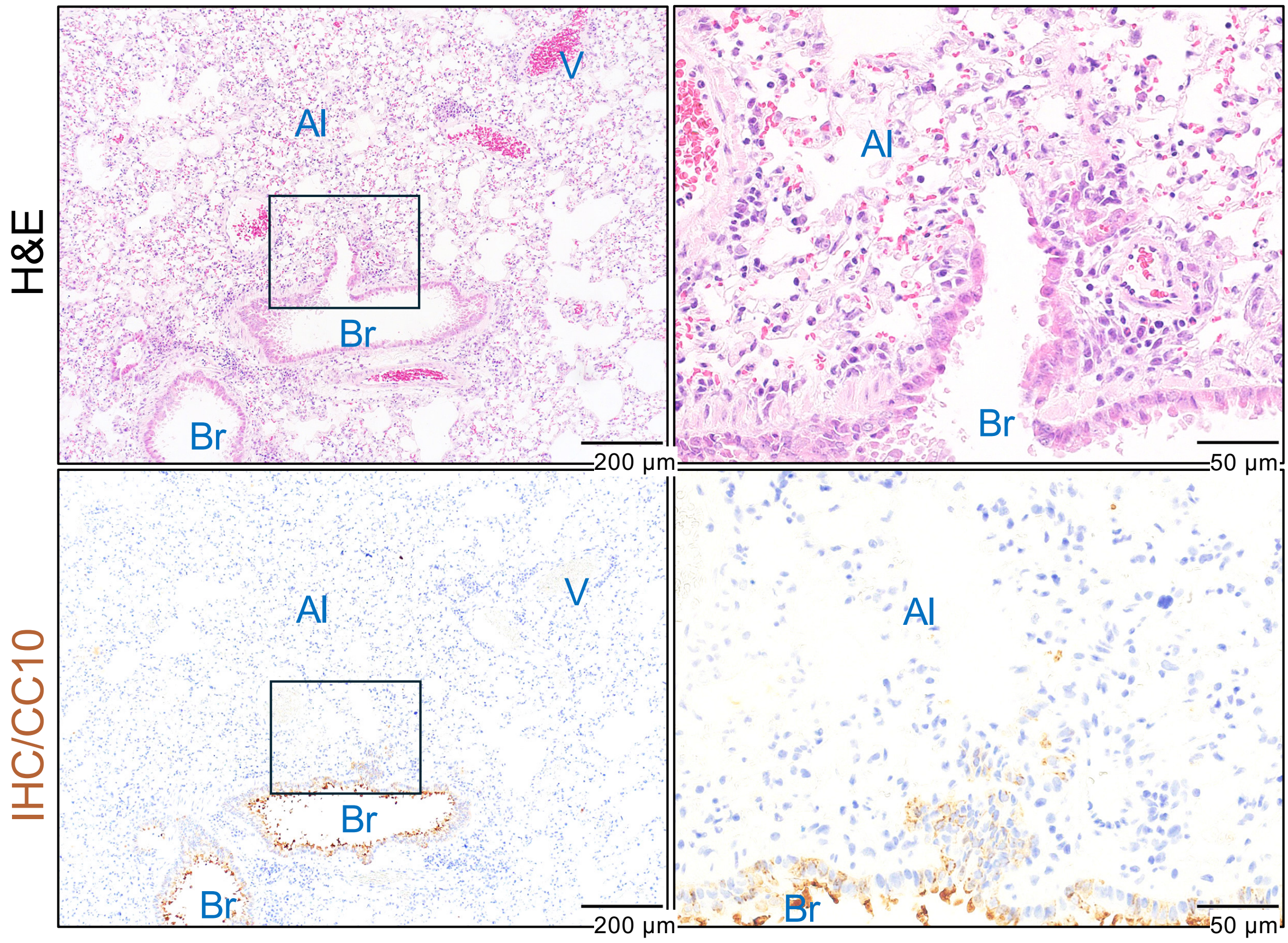

Fig. S6 Severe lung injury in an individual mouse with excessive weight loss after QHmusX infection. Representative image showing lung pathology from the individual in the OVA/Isotype group, as shown in Fig. 7C, who exhibited more than 25% body weight loss and reached a humane endpoint on day 6 p.i. Scale bars, 200 μm and 50 μm. H&E, hematoxylin and eosin staining; CC10, immunohistochemical staining for the CC10 antigen. Br, bronchiole; V, vessels; Al, alveoli.
